# Walking towards psychosocial well-being? Unveiling psychosocial impacts of a group-based walking program with and without cognitive enrichment in older adults—a mixed-methods randomized controlled trial

**DOI:** 10.7717/peerj.20569

**Published:** 2026-01-22

**Authors:** Pauline Hotterbeex, Jannique G.Z. van Uffelen, Julie Latomme, Melanie Beeckman, Stef Van Puyenbroeck, Sebastien Chastin, Greet Cardon

**Affiliations:** 1 Department of Movement Sciences, Leuven Brain Institute (LBI), Katholieke Universiteit Leuven, Leuven, Belgium; 2 Department of Movement and Sports Sciences, Ghent University Research for Aging Young (GRAY), Universiteit Gent, Gent, Belgium; 3 Expertise Center People & Society, Artevelde Hogeschool, Gent, Belgium; 4 School of Life Sciences, Glasgow Caledonian University, Glasgow, United Kingdom

**Keywords:** Mixed-methods, Randomized controlled trial, Psychosocial well-being, Older adults, Physical activity, Intervention, Cognitively enriched walking, Group-based

## Abstract

**Objectives:**

Evaluating the effects of a group-based cognitively enriched walking program (WALK+) and non-enriched walking program (WALK-only) on psychosocial well-being (as a secondary outcome) in community-dwelling older adults.

**Methods:**

A six-month randomized controlled trial was conducted, comparing WALK+, WALK-only and a passive control group. WALK+ and WALK-only involved two supervised group-based, and minimum one unsupervised walking session per week. Questionnaires on depressive symptoms, positive well-being, loneliness and social support measured psychosocial well-being at baseline, mid-intervention and post-intervention. Effects on these outcomes were assessed using linear mixed models with random intercepts. Self-perceived changes in psychosocial well-being were assessed post-intervention through a questionnaire and focus groups. Descriptive statistics were used for the questionnaire, and an inductive qualitative content analysis was conducted on the focus group data.

**Results:**

No significant intervention effects were found on depressive symptoms, positive well-being, loneliness and social support. Nevertheless, participants reported self-perceived improvements in psychological (40% of WALK+ and 56% of WALK-only participants) and social well-being (43% of WALK+ and 50% of WALK-only participants). The group sessions facilitated social connections, some lasting beyond the intervention period.

**Conclusions:**

Although no intervention effects were observed using standardized questionnaires, improvements in self-perceived psychosocial well-being suggest potential psychosocial benefits of WALK+ and WALK-only for older adults.

## Introduction

Both life expectancy and the proportion of older adults are projected to continue increasing (Eurostat, 2022; Eurostat, 2024), underscoring the importance of promoting healthy aging as a public health priority. In anticipation, the World Health Organization (WHO) developed a framework of healthy aging, comprising three components: functional ability, intrinsic capacity and the environment (World Health Organization (WHO), 2020). Functional ability entails the ability to build and maintain relationships, intrinsic capacity involves psychological capacity, and social support and relationships are part of the environment (World Health Organization (WHO), 2020). Furthermore, psychosocial well-being aligns with the general WHO definition of health: “a state of complete physical, mental and social well-being, not merely the absence of disease of infirmity” (World Health Organization (WHO), 2024). This definition emphasizes moving beyond the absence of ill-being such as depression or loneliness, to promote the presence of well-being, such as positive well-being or social support (Zhang & Chen, 2019). These constructs (depression, loneliness, positive well-being and social support) are collectively referred to as “psychosocial well-being” in the present manuscript.

Psychosocial well-being is associated with a range of physical and cognitive health outcomes in older adults. Depressive symptoms are linked to poor physical health, including an increased risk of cardiovascular disease and mortality (Gallo et al., 2005; Rajan et al., 2020), and can be a risk factor for cognitive decline as well as an early symptom of dementia (Livingston et al., 2020; Wiels, Baeken & Engelborghs, 2020). Conversely, positive well-being is related to a lower mortality risk (Zhang & Han, 2016). Furthermore, poor social support and loneliness are associated with a higher risk of mortality (Holt-Lunstad, Robles & Sbarra, 2017; Rico-Uribe et al., 2018), cardiovascular disease, stroke (Valtorta et al., 2016) and dementia (Kuiper et al., 2015; Penninkilampi et al., 2018).

Physical activity (PA) is a promising strategy to enhance psychosocial well-being. The association between PA and psychosocial well-being has been established in several observational studies in older adults. Higher levels of PA are inversely linked with depressive symptoms (Dubash, 2024; Levinsky, 2024; Schuch et al., 2018). However, this relationship may be bidirectional, as PA can reduce the risk of depressive symptoms, while depressive symptoms can lower future PA levels (Dubash, 2024; Levinsky, 2024). Similarly, a positive bidirectional relationship has been observed between PA and positive mental well-being (Bragina & Voelcker-Rehage, 2018; Ibáñez Román et al., 2023). For PA and loneliness some studies have found a inverse relationship, likely bidirectional (Pels & Kleinert, 2016b), while other studies have identified this relationship only with walking, and not with moderate-to-vigorous PA (MVPA) (McMullan et al., 2021; Shellito & Roldan, 2019). Additionally, PA has been positively linked to social support, potentially also in a reciprocal manner (Lindsay-Smith et al., 2018; Steinhoff & Reiner, 2024). Thus, evidence from observational research clearly points towards a bidirectional positive relationship between PA and psychosocial well-being.

However, the effects of PA interventions on psychosocial well-being, explored in experimental studies, are less conclusive. Whilst meta-analyses indicate that PA interventions can effectively reduce depressive symptoms in (older) adults with and without diagnosed depression (Hu et al., 2020; Noetel et al., 2024), few studies have focused specifically on positive well-being, with some indicating no and others indicating beneficial effects (Blodgett et al., 2022; Marquez et al., 2020; Windle et al., 2010). Results for social well-being are similarly mixed. Although some studies found no significant effects of PA on social support or loneliness in older adults (Düzel et al., 2022; Shvedko et al., 2018), others indicated that PA can alleviate loneliness, and increased social support may play a crucial role in this relationship (Ahn, Falk & Kang, 2024; Brady et al., 2020; Lindsay-Smith et al., 2018; Pels & Kleinert, 2016b). Consistent with other forms of PA, also walking, the most reported and preferred PA among older adults (Van Dyck et al., 2017), demonstrated positive effects on psychosocial well-being (Kelly et al., 2018). Furthermore, group-based PA (*e.g.*, walking) interventions in particular have shown benefits for psychosocial outcomes (Ahn, Falk & Kang, 2024; Bragina & Voelcker-Rehage, 2018; Kritz et al., 2021; Sebastião & Mirda, 2021). This could be explained by the social identity approach to health, suggesting that group activities create a sense of belonging to the group, and integrating the group membership into one’s social identity can elicit positive changes in psychosocial well-being (Christensen et al., 2006; Fransen et al., 2022; Stevens et al., 2017). Additionally, research has shown that PA in outdoor settings may have added benefits for health *versus* indoor PA (Noseworthy et al., 2023). In sum, interventional research on the psychosocial effects of PA has shown positive but somewhat inconclusive effects, with group-based walking as a promising avenue for future research.

In the scope of healthy cognitive aging, recent studies have focused on enriching PA with cognitive challenge in order to elicit larger effects on cognitive functioning compared to non-enriched PA (Gheysen et al., 2018; Rieker et al., 2022). However, few studies have assessed whether cognitively enriched PA also benefits psychosocial well-being. Those that have, focused only on outcomes such as depression, anxiety, stress, neuropsychiatric symptoms and quality of life, and found negligible effects (Gavelin et al., 2021; Zhu et al., 2016). Furthermore, these studies were mostly conducted in controlled laboratory settings, with interventions such as exergaming (Liao et al., 2021) or computerized cognitive training while cycling on a stationary bike (Pellegrini-Laplagne et al., 2023). To address the gap between laboratory and real-life settings, we recently developed a real-life, group-based cognitively enriched walking program together with academic experts and end-users (*i.e.,* older adults and walking coaches) (Hotterbeex et al., 2024; Marent et al., 2022). This program combines low-threshold cognitive tasks (*e.g.*, remembering a list of seasonal vegetables, solving riddles in order to find the correct route, or spotting things in the environment) with walking. Although the primary aim of the program was to prevent cognitive decline (of which the results have been published elsewhere: Hotterbeex et al., 2025), adding cognitive enrichment to PA in a group-based setting might foster interaction between group members. This could boost the sense of unity among group members (*i.e.,* similar to team-building activities) and the integration of the group into one’s social identity, thereby strengthening the effects on psychosocial well-being. On the other hand, performing cognitive tasks in a group setting may foster competition between participants, which is not always well-received (van Uffelen, Khan & Burton, 2017), or it may heighten individuals’ awareness of their own cognitive difficulties, potentially reducing the positive effects of PA on psychosocial well-being. Given the limited and inconclusive evidence, it is important to explore whether cognitively enriched PA holds benefits for psychosocial well-being and if these effects differ from those of standard PA without cognitive enrichment. While the exact mechanisms of cognitively enriched PA are still unclear due to limited research, directly comparing cognitively enriched PA and PA without enrichment allows us to begin disentangling whether cognitive enrichment enhances, diminishes, or has no additional impact on psychosocial well-being. This distinction is crucial for informing the design of future interventions that aim to support healthy ageing in general, including both cognitive and psychosocial health.

In this RCT, we examined the effects of the real-life, group-based cognitively enriched walking program we developed (WALK+) on psychosocial well-being in community-dwelling older adults, compared with a group-based walking program without enrichment (WALK-only) and a passive control condition (CONT). This paper addresses the following questions: (1) What are the effects of WALK+ on depressive symptoms, positive well-being, loneliness, and social support, compared with WALK-only and CONT?; and (2) What are the self-perceived effects of WALK+ on the psychosocial well-being, compared with WALK-only?

We hypothesized that WALK-only would increase psychosocial well-being if compared to CONT. The comparisons between WALK+ and CONT, and WALK+ and WALK-only were exploratory without predefined hypotheses, as little literature is available on this topic.

## Materials & Methods

### Design

This study uses secondary outcome data from a three-arm, parallel (1:1:1) randomized controlled trial (RCT) with a six-month intervention period (Hotterbeex et al., 2025). The RCT compared a cognitively enriched walking program (WALK+) with a walking program without enrichment (WALK-only) and a passive control condition (CONT). Data were collected at baseline, mid-intervention (after three months of intervention), and post-intervention (after six months of intervention) at two universities in Belgium (September 2022–April 2023). A mixed-methods triangulation design was adopted, collecting and analyzing both quantitative (baseline, mid- and post-intervention) and qualitative data (post-intervention only) (Creswell et al., 2003).

Baseline assessments were conducted prior to randomization. Included participants were randomly allocated to an intervention arm by a researcher not involved in the trial, stratified by location (Leuven or Ghent) and availability (two scheduling options for each location). The allocation sequence was generated by the Statistical Package for Social Sciences (SPSS) (IBM Corp, 2021). Participants and researchers were informed of the allocation after the baseline measurements, ensuring double-blind data collection at baseline. Subsequent measurements were nonblinded. Only participants of WALK+ and WALK-only were invited to participate in the evaluation of self-perceived effects, which included a questionnaire and a focus group.

A *post-hoc* power analysis conducted for the secondary outcomes with the software G*Power 3.1.9.7 for a repeated measures ANOVA with within-between interactions (null hypothesis: there is no interaction effect between time and condition) indicated that the sample size (*n* = 145 analyzed) with three groups and three measurements per group, could detect Cohen’s d effect sizes of 0.29 with 80% power at *α* = 0.05 (Faul et al., 2007). This approach was used as a conservative and commonly accepted approximation for estimating detectable effect sizes in longitudinal designs, given that G*Power currently does not support power calculations for linear mixed models.

The RCT was registered at ClinicalTrials.gov (NCT05500183) and was approved by the Ethical Committee of the Faculty of Psychology and Educational Sciences of Ghent University (2022-065 and 2023-029) and the Committee of Medical Ethics of the Ghent University Hospital (B6702022000564). All participants provided written informed consent. The reporting in this manuscript adheres to the CONSORT reporting guidelines (Schulz, Altman & Moher, 2010).

### Participants

Participants for the RCT were recruited from June-October 2022 by distributing flyers through various channels, including organizations for older adults, local service centers, community health centers and social media. On a study website, those interested could find information on the study and a contact form. The primary outcome of this RCT was cognitive functioning, and community-dwelling adults aged 65+ years were excluded in case of: diagnosis of a neurodegenerative or psychiatric disorder, ongoing recovery from severe brain injury, current or past addiction to substances, mobility limitations, parental history of young-onset dementia and unavailability during ≥1 month of the intervention. Participants were screened by P.H., M.B., or J.L. for these criteria during a telephone call and enrolled when eligible. Additional baseline screening was conducted face-to-face for mild cognitive impairment (MCI) (Montreal Cognitive Assessment (MoCA), cut-off 23/30) (Carson, Leach & Murphy, 2018; Nasreddine et al., 2005) and severe depressive symptoms (Beck Depression Inventory-II (BDI-II), cut-off 29/63) (Balsamo et al., 2018; Beck, Steer & Brown, 1996). While the standard MoCA cut-off score is 26/30, this study adopted a lower threshold of 23/30 to reduce the risk of false positives (Carson, Leach & Murphy, 2018). This decision was supported by healthcare professionals working in (neuro)geriatric settings who were consulted in preparation for this study.

### Interventions

WALK+ and WALK-only lasted six months, with participants of both conditions attending two 60–90 min outdoor group sessions per week, primarily in green spaces. Additionally, participants were instructed to perform at least one unsupervised session per week. Each group was supervised by a walking coach who used a manual to standardize the sessions (the walking was instructed to be brisk, which corresponds to a moderate intensity) while also allowing some flexibility in location and route (File S4). Although the duration of the sessions did not progressively increase, the coaches were instructed to try to increase the walking pace. The key difference between WALK+ and WALK-only was the cognitive enrichment, only present in WALK+, consisting of 15–20 min of cognitive tasks per 30 min of walking. These tasks targeted a variety of cognitive functions, including memory, attention, and executive functioning, and were co-designed with academic experts and end-users. Further details are provided in our published manuscripts on the development study (Marent et al., 2022) and pilot study (Hotterbeex et al., 2024). The development included a three-round Delphi study (*n* = 34 academic experts) and a survey study with older adults and walking coaches (*n* = 535) (Marent et al., 2022). The pilot study was conducted in two phases due to COVID-19 and involved walk-along interviews (*n* = 163) and a group-based pilot (*n* = 19). The cognitively enriched walking program was refined based on the results of this pilot study (Hotterbeex et al., 2024). The cognitive tasks were performed in groups, pairs, or individually, with instructions for coaches provided in the manual to ensure consistency. Participants of the WALK+ program also received practice cards with simplified explanation of the cognitive tasks to facilitate the unsupervised sessions (Fig. S1). In total, there were eight walking groups, each consisting of approximately ten participants: two WALK+ groups in Leuven, two WALK+ groups in Ghent, two WALK-only groups in Leuven, and two WALK-only groups in Ghent. The walking programs were compared to a passive control condition (CONT) for which participants were instructed to continue their daily activities.

### Measurement materials

#### Sociodemographics and PA

At baseline, age, sex, educational level, country of birth and marital status were collected. Baseline PA was measured using an ActiGraph GT3X+ worn on the hip for seven consecutive days before the start of the intervention (ActiGraph, Pensacola, FL, USA). Data were collected at 100 Hz, and processed in the ActiLife 6.13.4 software. The Choi et al. (2011) algorithm was used to define (non)-wear time, and only participants with ≥4 days with ≥10 h of wear-time were included (Migueles et al., 2017). Data were processed in 5 s epochs to align with the processing in the development of the cut-point for MVPA (1,013 counts/min) that we used (Barnett et al., 2016).

#### Outcome measures—quantitative

Psychosocial well-being was seen in this study as the combination of psychological and social well-being. Psychological well-being refers to an individual’s mental and emotional state (here: depressive symptoms and positive well-being), while social well-being refers to an individual’s social relationships and connectedness (here: social support and loneliness).

##### Depressive symptoms (baseline, during, post-intervention).

Depressive symptoms were measured using the Beck Depression Inventory (BDI-II) (Beck, Steer & Brown, 1996). This 21-item self-report questionnaire uses a Guttman scale, with a total score of 0–63, and scores of 0–13 indicating minimal, 14–19 mild, 20–28 moderate and 29–63 severe depressive symptoms (Balsamo et al., 2018). Cronbach’s Alphas ranged from 0.81–0.86.

##### Positive well-being (baseline, during, post-intervention).

The score of the 14-item Warwick Edinburgh Mental Well-being Scale (WEMWBS) (Stewart-Brown et al., 2011) ranges from 14–70 and higher scores indicating better well-being. The WEMWBS has a good content validity and is sensitive to change (Stewart-Brown et al., 2011). Cronbach’s Alphas of 0.90–0.92 were observed.

##### Loneliness (baseline, during, post-intervention).

The 11-item de Jong-Gierveld Loneliness Scale (De Jong-Gierveld & Kamphuls, 1985) assessed loneliness using items rated on a five-point Likert scale, resulting in a total score ranging from 0–11 with a higher score indicating higher loneliness. Its validity and reliability have been shown to be sufficient (De Jong-Gierveld & van Tilburg, 1987). Cronbach’s Alphas of 0.88–0.89 were observed.

##### Social support (baseline, during, post-intervention).

The 6-item Social Support List—Interaction (SSL12-I) (van Eijk, Kempen & van Sonderen, 1994) evaluated items on a four-point rating scale. The total score ranges from 12–48 with higher scores indicating more social support. Its psychometric properties are satisfactory (Kempen & van Eijk, 1995). Within our sample, Cronbach’s Alphas ranged from 0.88–0.90.

##### Self-perceived changes in psychosocial well-being (post-intervention).

Participants in the WALK+ and WALK-only groups rated perceived changes in psychological and social well-being on a five-point scale from “Much worse psychological/social well-being (1)” to “Much better psychological/social well-being (5)”, with “Neither better nor worse (3)” as middle point.

#### Outcome measures—qualitative

A generic qualitative approach was adopted, which allows for methodological flexibility and focuses on the understanding of participants’ experiences (Ellis & Hart, 2023; Savin-Baden & Howell Major, 2013). Only WALK+ and WALK-only participants were part of the qualitative examination.

##### Self-perceived psychosocial well-being (post-intervention).

Two sources of qualitative data were analyzed: *(1) open-ended questionnaire responses* and *(2) focus group discussions*.

First, space was provided for participants to explain their responses on the rating scales in the questionnaire on self-perceived changes in psychosocial well-being.

Second, to gain a deeper insight into their experiences, participants of WALK+ and WALK-only were invited to discuss perceived changes in psychosocial well-being during focus groups. The focus groups were part of a larger process evaluation, but for the present manuscript, only data about the participants’ self-perceived changes were analyzed. P.H. moderated the focus groups using a semi-structured interview guide (File S5), and M.B. observed and took notes. Eight focus groups were organized (one for each walking group in both the WALK+ and WALK-only condition), with on average seven participants present (range: 5–9). The average duration of the whole focus groups (*i.e.,* including all questions) was 86 min for WALK+ and 60 min for WALK-only. All focus groups were audio recorded, with the participants’ consent.

### Statistical analysis

#### Quantitative

All inferential analyses were conducted in R version 4.2.2. (R Core Team, 2022), descriptive analyses were conducted in SPSS (IBM Corp, 2021). Baseline sociodemographic characteristics were compared using Chi-squared tests for nominal variables and Kruskal-Wallis tests for ordinal and non-normally distributed variables.

##### Questionnaires on depressive symptoms, positive well-being, loneliness and social support.

Linear mixed models with random intercepts were conducted using the packages *lme4* (version 1.1-33), *lmerTest* (version 3.1-3) and *emmeans* (version 1.8.9). Condition (WALK+, WALK-only, CONT) was included as a level 2 predictor (between-subjects), and time (baseline, mid-intervention, post-intervention) as a level 1 predictor (within-subjects). Both variables were included in the model as categorical factors. The model formula can be found in File S6. All pairwise interaction effects of interest (WALK+ *vs.* WALK-only x time, WALK+ *vs.* CONT x time, and WALK-only *vs.* CONT x time) were estimated using the *emmeans* package with the *contrast* function. Both crude models and adjusted models, corrected for age, sex, marital status and baseline PA were run. As there were no differences in interaction effects between the crude (Table S1) and adjusted models, the adjusted models were reported. Cross-level interactions were assessed to determine if changes over time differed by condition. Significant main or interaction effects were followed by *post-hoc* pairwise comparisons using the multivariate t correction for multiple testing to limit the risk of type 1 errors. It applies the multivariate t distribution, incorporating the same covariance structure as the estimates to determine the adjustment (Lenth et al., 2025). The normality of residuals was visually checked using Q-Q plots and histograms. If non-normality was detected, analyses were conducted with and without extreme outliers. As results were consistent, the analyses including residual outliers are reported. The analysis followed an intention-to-treat approach, including all participants in the analysis.

##### Questionnaire on self-perceived change in psychosocial well-being.

These data were analyzed using descriptive statistics.

### Qualitative

The focus groups were transcribed verbatim, and sections related to self-perceived changes in psychological and social well-being were extracted. An inductive qualitative content analysis (Hsieh & Shannon, 2005) was performed using Nvivo software (Lumivero, 2023), separately on the focus group transcripts and the qualitative responses in the questionnaire on self-perceived changes, allowing themes to emerge directly from the data. The data were read repeatedly for immersion, and coded line by line. Codes were then revised and sorted into meaningful categories and subcategories by P.H. Initial categories were discussed with J.L. and J.v.U., and adapted accordingly. The analysis was iterative, moving back and forth between the different phases of the analysis (*i.e.,* immersing in data, coding, and sorting codes into categories).

## Results

**Figure 1:**
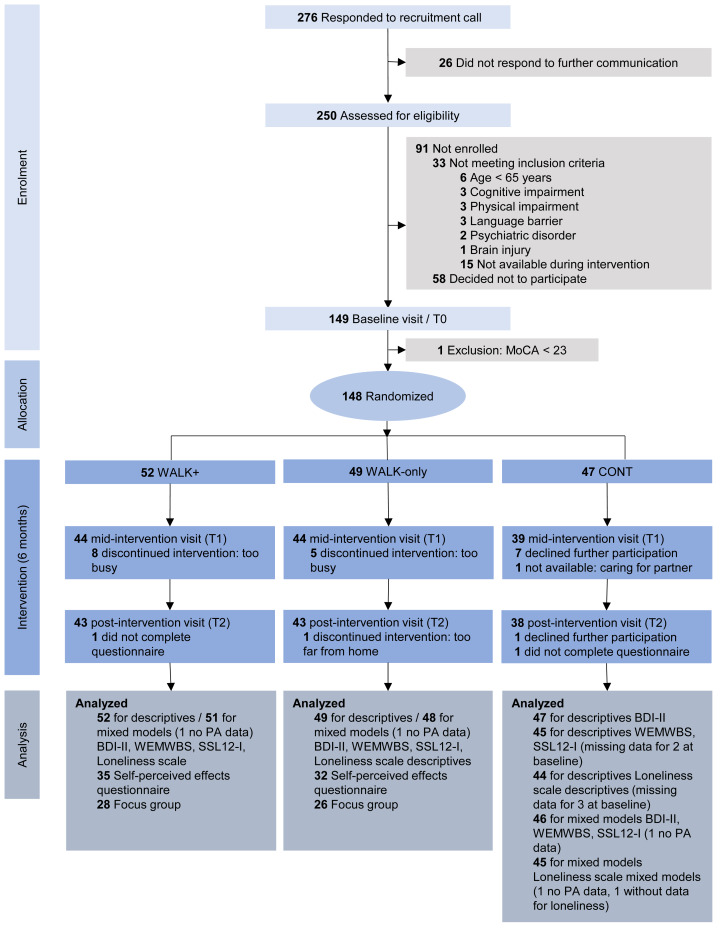
Study flowchart. One participant that discontinued WALK+ was included in the analyses of self-perceived changes as we deem this information relevant.

One hundred forty-eight participants were randomized to one of the three conditions (WALK+, WALK-only or CONT). Post-intervention, 125 participants remained in the study, indicating a drop-out of 23 participants (16%) during the intervention period. On average, participants were 70 years old at baseline (range: 65–85), and 72% (*n* = 107) were female. A large proportion was highly educated (47% with Master’s degree), and the majority was in a relationship (63%). At baseline, the WALK+, WALK-only and CONT conditions were comparable in terms of sociodemographics. See Fig. 1 and Table 1 for the participant flow and sociodemographic characteristics, respectively.

**Table 1:** Sociodemographic characteristics by condition and for the total sample.

Variable	% (*n*) unless otherwise stated				*p*
	WALK+ (*n* = 52)	WALK-only (*n* = 49)	CONT (*n* = 47)	Total (*n* = 148)	
Age, mean (range)	70 (65–85)	69 (65–79)	69 (65–84)	70 (65–85)	0.80
Sex					0.78
Female	69 (36)	76 (37)	72 (34)	72 (107)	
Male	31 (16)	24 (12)	28 (13)	28 (41)	
Country of birth					0.91
Belgium	94 (49)	96 (47)	96 (44)	95 (140)	
Other	6 (3)	4 (2)	4 (2)	5 (7)	
Missing	0 (0)	0 (0)	0.02 (1)	0.01 (1)	
Level of education					0.91
Left formal education ≤16 y	2 (1)	0 (0)	2 (1)	1 (2)	
Left formal education at 17–18 y	14 (7)	14 (7)	21 (10)	16 (24)	
Undergraduate degree or equivalent	39 (20)	37 (18)	32 (15)	36 (53)	
Master’s degree or higher	46 (24)	49 (24)	45 (21)	47 (69)	
Marital status					0.54
Single	25 (13)	31 (15)	26 (12)	27 (40)	
Widow(er)	14 (7)	4 (2)	13 (6)	10 (15)	
In a relationship	62 (32)	65 (32)	62 (29)	63 (93)	
MVPA (min/day), median (inter-quartile range)	101.29 (41.01)	77.55 (35.56)	83.02 (37.18)	85.24 (41.98)	0.08

**Notes.**

MVPA, moderate-to-vigorous physical activity.

### Quantitative

#### Depressive symptoms, positive well-being, loneliness and social support

Descriptive statistics for psychosocial well-being outcomes are displayed in Table 2. Descriptively, mean scores remained relatively stable over time and across groups. There were no statistically significant main effects of time for any of the conditions (WALK+, WALK-only and CONT), nor any statistically significant main effects of condition^1^ 1After removal of two residual outliers for BDI-II (depressive symptoms), the main effect of WALK+ *vs.* CONT was significant (*p* = 0.04). However, no significant *post-hoc* pairwise comparisons between WALK+ and CONT were found at any of the time points.at any of the time points (pre-intervention, mid-intervention, and post-intervention; Table S2). Furthermore, no statistically significant interaction effects between time and condition were found for any of the outcomes, indicating that the change over time did not differ between conditions (Table 3). Females reported higher levels of perceived social support than males. Apart from this, none of the covariates (age, sex, civil status, and baseline PA) were significant across outcomes (Table S3).

**Table 2:** Psychosocial well-being by condition and for the total sample.

	Descriptives (*n* = 148) M (SD)		
	Pre	3 m	6 m
Depressive symptoms (BDI-II), min. 0–max. 63, higher scores indicate more depressive symptoms			
WALK+	4.75 (3.98)	4.80 (4.72)	5.35 (4.63)
WALK-only	5.63 (4.65)	5.84 (5.64)	5.86 (5.71)
CONT	6.45 (4.48)	6.26 (5.06)	5.78 (4.73)
Total	5.58 (4.40)	5.60 (5.15)	5.66 (5.03)
Positive well-being (WEMWBS), min. 14–max. 70, higher scores indicate higher positive well-being^a^			
WALK+	56.71 (6.34)	56.33 (6.54)	55.84 (7.70)
WALK-only	53.84 (6.07)	54.02 (6.50)	54.19 (6.17)
CONT	54.49 (7.17)	53.51 (6.19)	54.47 (6.55)
Total	55.06 (6.60)	54.68 (6.49)	54.85 (6.83)
Loneliness (De Jong-Gierveld), min. 0–max. 11, higher scores indicate more loneliness^b^			
WALK+	3.35 (3.16)	3.18 (3.16)	3.53 (3.36)
WALK-only	3.29 (3.26)	3.95 (3.78)	3.65 (3.50)
CONT	3.98 (3.69)	4.05 (3.47)	3.89 (3.56)
Total	3.52 (3.35)	3.71 (3.48)	3.68 (3.45)
Social support (SSL12-I), min. 12–max. 48,higher scores indicate more social support^a^			
WALK+	30.31 (5.54)	30.60 (5.63)	30.21 (4.98)
WALK-only	29.94 (5.43)	29.52 (5.33)	30.16 (6.83)
CONT	28.76 (5.85)	28.62 (5.19)	28.45 (5.92)
Total	29.71 (5.60)	29.63 (5.42)	29.65 (5.97)

**Notes.**

BDI-II, Beck Depression Inventory-II; WEMWBS, Warwick Edinburgh Mental Wellbeing Scale; SSL12-I, Social Support List-Interaction.

a Missing data at baseline *n* = 2.

b Missing data at baseline *n* = 3.

**Table 3:** Intervention effects on psychosocial well-being (linear mixed models).

	WALK+ *vs.* WALK-only				WALK+ *vs.* CONT				WALK-only *vs.* CONT			
	*β*	df	95%CI	*p*	*β*	df	95%CI	*p*	*β*	df	95%CI	*p*
Depressive symptoms (BDI-II) *n* = 145												
Pre *vs.* 3 m	0.25	258	−1.48;1.98	0.78	0.04	260	−1.75;1.82	0.97	−0.21	258	−2.00;1.58	0.82
Pre *vs.* 6 m	−0.21	258	−1.96;1.54	0.81	−1.12	261	−2.93;0.68	0.22	−0.91	259	−2.72;0.90	0.32
Positive well-being (WEMWBS) *n* = 145												
Pre *vs.* 3 m	0.76	252	−1.20;2.71	0.45	−0.56	255	−2.59;1.47	0.59	−1.31	253	−3.35;0.72	0.21
Pre *vs.* 6 m	0.97	252	−1.00;2.94	0.33	1.11	254	−0.94;3.15	0.29	0.14	252	−1.91;2.19	0.89
Loneliness (De Jong-Gierveld) *n* = 144												
Pre *vs.* 3 m	0.54	244	−0.33;1.42	0.22	0.12	247	−0.79;1.04	0.79	−0.42	245	−1.33;0.49	0.37
Pre *vs.* 6 m	−0.17	244	−1.04;0.71	0.71	−0.51	246	−1.43;0.40	0.27	−0.35	245	−1.26;0.57	0.46
Social support (SSL12-I) *n* = 145												
Pre *vs.* 3 m	−0.26	255	−2.06;1.55	0.78	0.35	258	−1.52;2.22	0.71	0.61	256	−1.27;2.48	0.53
Pre *vs.* 6 m	0.75	255	−1.07;2.57	0.42	0.32	258	−1.57;2.21	0.74	−0.44	257	−2.33;1.46	0.65

**Notes.**

*β* represents the estimate for the interaction effect between Time (Pre *vs.* 3 m and Pre *vs.* 6 m) and Condition (WALK+ *vs.* WALK-only, WALK+ *vs.* CONT and WALK-only *vs.* CONT), which indicates estimated difference between conditions in change from baseline to each follow-up time point; BDI-II, Beck Depression Inventory-II; WEMWBS, Warwick Edinburgh Mental Wellbeing Scale; SSL12-I, Social Support List-Interaction.

#### Self-perceived effects on psychosocial wellbeing

**Figure 2:**
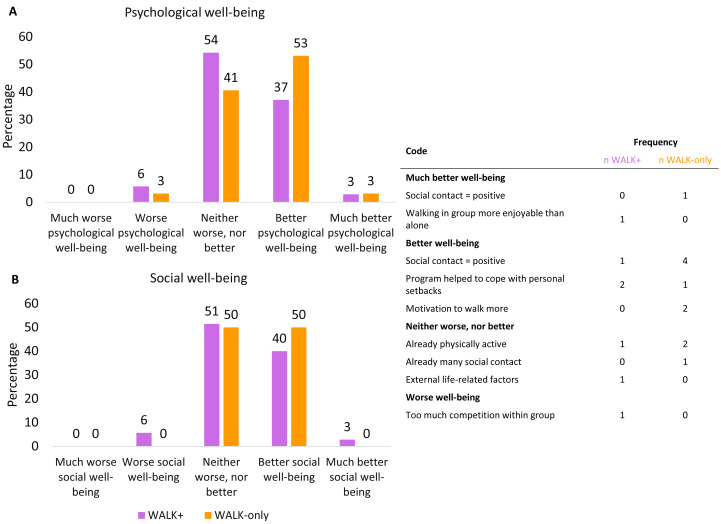
Self-perceived changes in psychological and social well-being. (A) Psychological well-being. (B) Social well-being. The table is for psychological and social well-being together. Data for: WALK+ *n* = 35; WALK-only *n* = 32.

Post-intervention, 81% of the WALK+ (*n* = 35) and 74% of the WALK-only (*n* = 32) participants completed the questionnaire on self-perceived changes. Forty-five percent of WALK+ and 41% of WALK-only reported no change in psychological well-being, while 40% of WALK+ and 56% of WALK-only participants reported an improvement (*i.e.,* better or much better psychological well-being). For social well-being, 51% of WALK+ and 50% reported no change, while 43% of WALK+ and 50% of WALK-only reported an improvement (*i.e.,* better or much better social well-being). For psychological well-being, 6% of WALK+ and 3% of WALK-only reported a decrease. For social well-being, this was 6% of WALK+ and 0% of WALK-only (Fig. 2).

### Qualitative

#### Reasons for reported change in self-perceived psychosocial well-being

Those reporting no change attributed this to already being physically active (*n* = 3), having sufficient social contacts (*n* = 1), or having received a serious health diagnosis (*n* = 1). Those reporting better well-being, mentioned positive interactions with the walking group (*n* = 5), that the program helped to cope with personal setbacks (*n* = 3), or increased walking prompted by the program (*n* = 2). Those reporting much better psychological and/or social well-being, attributed this to the positive social contacts (*n* = 1) and finding walking in group more enjoyable than walking alone (*n* = 1). Participants reporting worse psychological and social well-being, cited too much competition within the walking group (Fig. 2).

#### Focus groups

**Figure 3:**
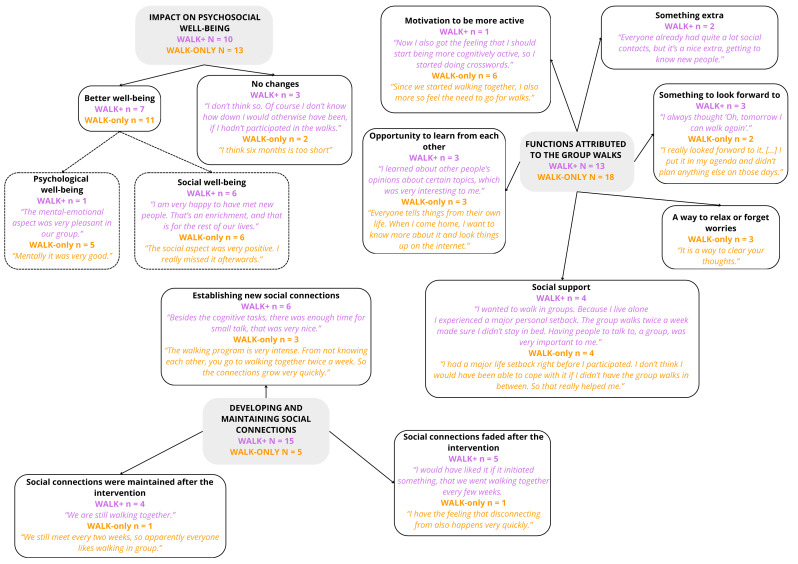
Visual representation of the (sub)categories defined in the content analysis, including counts and quotes.

Of participants still in the study post-intervention, 64% (28/44) of WALK+ and 60% (26/43) of WALK-only participants attended the focus groups. In the content analysis, three main categories were developed: *impact on psychosocial well-being*, *functions attributed to the group walks*, and *development and maintenance of social connections*. In Fig. 3, the themes and subthemes, including frequencies and representative quotes can be found. Overall, findings show similar patterns across WALK+ and WALK-only conditions, with notable differences highlighted in the summary below.

##### Impact on psychosocial well-being.

Participants were generally positive about the psychosocial effects of the intervention. Some indicated that it was good for their psychological well-being, it made sure they were off for a good start of the day. Others mentioned having pleasant, new social contacts as the most important benefit of the intervention. Nevertheless, some participants indicated that they were not aware of any psychosocial benefits of the intervention.

##### Functions attributed to the group walks.

The group walks served various roles for different participants. First, it could be a motivator to be more active in their daily life, this was mentioned mainly by the WALK-only participants. Participants in the WALK-only condition started enjoying walking more, even on their own, which they did not enjoy before the program. They also felt activated after the group walk in the morning, and felt they had more energy. In addition, participants in the WALK+ condition indicated that they were motivated by walking in group instead of alone. The program was also a motivator to be more cognitively active. Second, the group walks were an opportunity to learn about and from each other. This was not only a result of the cognitive tasks, but also more generally of the social contact, as participants of both conditions mentioned this. Third, the group walks were perceived as a form of social support. This was the case for participants who otherwise would have to walk alone, and in particular for two participants, one in the WALK+ and one in the WALK-only condition, who experienced a major personal loss during and right before the intervention. Fourth, the walks provided a way to relax or forget worries for WALK-only participants, enabling participants to escape the stresses of daily life and clear their minds. Fifth, participants perceived the group walks as something to look forward to, and for some the dates of the walks were fixed in their agenda. Last, for some of the WALK+ participants, the walks were simply an enjoyable addition to their usual social activities.

##### Establishing and maintaining social connections.

In both conditions, new social connections were established, but this theme was mentioned remarkably more times by WALK+ participants. They mentioned feeling comfortable to discuss anything with each other, with no topics being off-limits. They shared that during the intervention, they established a genuine connection and came to care about each other. Although there were mentions of social connections fading after the intervention ended, we are aware of three WALK+ and three WALK-only groups that are still (occasionally) walking together. Some participants wondered if six months is sufficient to establish long-lasting social connections. Participants of the WALK+ group who discontinued their walks shared their disappointment, hoping to continue walking together. As mentioned before, other groups continued their walks, and some even invited their partners or friends to participate.

## Discussion

The aim of this mixed-method study was to explore the effects of a group-based cognitively enriched walking program (WALK+) compared to a walking program without enrichment (WALK-only) and a passive control group (CONT), on psychosocial well-being (as a secondary outcome) of community-dwelling older adults.

In contrast to our hypothesis, neither WALK+ nor WALK-only resulted in significant improvements in psychosocial well-being compared to CONT, as measured by standardized questionnaires. This was particularly surprising for depressive symptoms, as previous intervention studies have supported the positive impact of PA on reducing depressive symptoms, in both clinical (Noetel et al., 2024) and general populations (Hu et al., 2020). It was however mentioned that there is high heterogeneity between different studies, and that publication bias may be present (Hu et al., 2020). Additionally, our findings do not align with the limited but suggestive literature on the potential benefits of PA on positive well-being (Blodgett et al., 2022; Marquez et al., 2020; Windle et al., 2010). Furthermore, while some have reported positive effects of PA on loneliness and social support (Ahn, Falk & Kang, 2024; Brady et al., 2020; Lindsay-Smith et al., 2018; Pels & Kleinert, 2016b), our results were more consistent with research indicating no significant effects in these areas (Düzel et al., 2022; Shvedko et al., 2018). These results do align with previous studies not finding a significant impact of cognitively enriched PA on depressive symptoms (Gavelin et al., 2021; Zhu et al., 2016). Our study is, to our knowledge, one of the first to explore the effects of a cognitively enriched PA intervention on positive well-being, social support, and loneliness.

There were no significant effects on the outcomes mentioned above. However, a substantial part of the participants reported a perceived improvement in their psychological and social well-being following the intervention. Specifically, 40% of WALK+ and 53% of WALK-only perceived (much) better psychological well-being, while 43% and 50% of the respective conditions reported (much) better social well-being. These self-perceived improvements were further supported by qualitative data from the focus groups, where participants reported various positive aspects of the group walks, such as social support, motivation to be more active, and the establishment of social connections, some that lasted even after the end of the intervention. However, these impressions should be interpreted with caution, as they are not aligned with the standardized assessments. Moreover, only participants in the WALK+ and WALK-only conditions were asked about self-perceived improvements, and thus, no comparison with those in the CONT condition was possible.

Interestingly, distinct psychosocial constructs such as depressive symptoms, positive well-being, loneliness and social support were not explicitly mentioned during the focus groups. This was in line with the mixed-methods study of Lindsay-Smith et al. (2018), where participants also reported the social benefits of PA in group (*e.g.*, social connectedness, development of friendships), but did not specifically mention the social constructs being measured (*i.e.,* social support and loneliness). Moreover, participants in our study mainly spoke about social and psychological well-being when they perceived the program as a means to cope with major personal losses. Likewise, Lindsay-Smith et al. (2018) indicated that, particularly when experiencing major life events or setbacks, group membership can be experienced as a “safety net” or a means to forget worries.

A slightly higher proportion of the WALK-only condition indicated improvements in both psychological and social well-being compared to the WALK+ condition. Two participants of WALK+ (both in different walking groups) indicated negative changes in social and psychological well-being, for one this was due to perceived competition during the cognitive tasks. Before the intervention start, coaches in the WALK+ condition were instructed to carefully include competition as it can be motivating for some, but may have negative impacts on others (van Uffelen, Khan & Burton, 2017). Therefore, it is possible that some participants, driven by their competitive nature, may have influenced the group dynamics in the WALK+ condition. The focus groups did not reveal a clear reason for the differences between WALK+ and WALK-only, and the observed discrepancy in perceived effects were not statistically significant. Possibly, the discrepancy is due to participants in WALK+ having less time for small talk, as a significant portion of the WALK+ sessions was dedicated to performing cognitive tasks.

Several factors could explain the lack of significant quantitative findings for depressive symptoms, positive well-being, loneliness and social support. First, the WALK+ program did not lead to increased MVPA, while the WALK-only program did (+12 min MVPA/day) (Hotterbeex et al., 2025). The absence of an increase in PA in the WALK+ group may have contributed to the lack of measurable effects on psychosocial well-being. Additionally, it could be argued that walking was not intensive enough to elicit effects. However, lower-intensity activities such as walking have been shown to benefit psychosocial well-being through mechanisms beyond physiological and brain changes (Bragina & Voelcker-Rehage, 2018; Düzel et al., 2022; Kelly et al., 2018; Pels & Kleinert, 2016a). Furthermore, moderate intensity of PA offers no certainty of effects, as Düzel et al. (2022) did not find effects on psychosocial well-being of an individually tailored, progressively increasing moderate intensity program.

The selectivity of our sample may also have influenced the results. Participants were relatively physically active, and had high psychosocial well-being at baseline, leaving little room for improvement (Düzel et al., 2022; Harris, 2018).

Additionally, an intervention duration of six months may not be sufficient to produce significant changes. Lindsay-Smith et al. (2018) found a significant reduction in loneliness after one year, and suggest that a longer intervention may be needed for changes in social support. In a meta-analysis, Park, Han & Kang (2014) showed significant effects on depressive symptoms after only three months, but this may have been influenced by the inclusion of participants with minor depression at baseline. For positive well-being, Harris (2018) found an improvement after an 8-month-long intervention. This evidence suggests that changes in these constructs may require longer periods to manifest.

Finally, the measurements used in this study may not have been sensitive enough to detect changes in psychosocial well-being. Although many participants reported self-perceived improvements (in the questionnaire and focus groups), this may not have been captured by the standardized questionnaires used. However, while qualitative and self-reported data provide valuable context, these findings should be interpreted as exploratory. The absence of effects on standardized questionnaires may be explained by floor effects in the questionnaires for depressive symptoms and loneliness, as the participants in the present study had very low scores at baseline. Ceiling effects for positive well-being and social support are less supported in our data. Furthermore, the measures used in this study may not have covered aspects of psychosocial well-being that did change. For example, future research could consider including measures of social identity, as group identification may play a role in improving psychosocial well-being with group-based interventions (Haslam et al., 2009).

### Strengths and limitations

The mixed-methods design of this study is a strength. It allowed us to delve deeper into the intervention effects than simply conclude that the interventions were not effective, based on the standardized questionnaires. It ensured a broad exploration, allowing the detection of effects on other constructs than those measured with standardized questionnaires. Furthermore, conducting an intention to treat analysis prevented bias due to participant drop-out (McCoy, 2017). Additionally, using linear mixed models allowed us to include all available data points, also from participants with missing values. Lastly, the drop-out rate was relatively limited, 126 of 148 participants (*i.e.,* 85%) completed the post-intervention measurement.

This study also had its limitations. First, 72% of the sample was female, and therefore, the findings of this study may be sex-specific. Next, a large proportion of the sample was highly educated, possibly limiting the generalizability to participants with lower levels of education. The sample was also relatively physically active at baseline, with good psychosocial well-being, limiting the room for improvement.

In addition, although the selected outcome measures (BDI-II, WEMWBS, De Jong-Gierveld Loneliness Scale, and SSL12-I) are validated for use in healthy populations, the generally high baseline levels of psychosocial well-being in our sample may have introduced a ceiling effect for the WEMWBS and SSL12-I, and a floor effect for BDI-II and the De Jong-Gierveld Loneliness Scale. This could limit the sensitivity of these instruments to detect further improvements, particularly in participants who already had high well-being. A visual inspection of the data pointed towards possible floor effects for depressive symptoms and loneliness, while ceiling effects for positive well-being and social support are less supported. As such, the lack of observed changes should be interpreted with caution, and future studies might consider stratifying participants by baseline well-being or using measures with greater sensitivity to subtle changes in high-functioning individuals. However, as the examination of the effects of WALK+ was exploratory, theoretically, a decline in psychosocial well-being was also possible, and this could have been detected by the measures used.

Furthermore, only the WALK+ and WALK-only conditions were asked about self-perceived changes in psychosocial well-being, and thus no comparison with the CONT condition was possible for these outcomes. This leaves it uncertain whether similar improvements might have been reported by the participants in the CONT condition if they had been asked the same questions. In addition, not being able to make a comparison with a social activity only control condition limits our ability to attribute the self-perceived improvements in psychosocial well-being to (cognitively enriched) PA.

With our sample, effect sizes of 0.29 could be detected with a power of 80%, indicating that smaller effects might not have been identified. Furthermore, the examined programs were not specifically developed to improve psychosocial well-being, which may have limited the potential effects. However, it is important to explore potential benefits of interventions beyond their primary outcome, as cost-effective interventions are needed to promote healthy aging on a population level.

### Future research directions

Future research should aim to include more diverse samples. Specifically, recruiting individuals with lower baseline psychosocial well-being (*e.g.*, those experiencing loneliness, low positive well-being, low social support, or clinical levels of depression), and those who are less physically active may reduce ceiling or floor effects and increase the likelihood of detecting intervention effects.

Additionally, future studies could tailor interventions more specifically toward psychosocial outcomes, for example, by integrating structured social interaction components or emotional regulation strategies alongside physical and cognitive tasks. This may enhance the relevance and impact of the intervention on psychological and social well-being.

A potential avenue for future cognitively enriched PA interventions, mitigating the challenge of negative competition, could be reminiscence-based activities (*i.e.,* participants recall and discuss past life experiences). Reminiscence activity has shown the potential to improve psychosocial well-being and cognitive functioning, though the most promising effects up until now have been observed in individuals with dementia (Dinius et al., 2023). It is possible—and feasible—to combine reminiscence activities with walking in older adults, but no firm conclusions have yet been drawn regarding the effects of this combination on cognitive functioning or psychosocial well-being (Croff et al., 2024; Dinius et al., 2023).

Other outcomes should also be considered. For example, although anxiety was not assessed in the current study, it could emerge as a relevant outcome, particularly in response to the cognitive demands or group-based nature of the intervention. Future research may consider assessing such responses to better understand the broader psychosocial effects of interventions like WALK+. Furthermore, future studies on cognitively enriched PA interventions should continue to include psychosocial well-being as an outcome in order to get a clearer image of whether combined interventions can improve psychosocial well-being.

To better understand mechanisms at play, future research should also explore the dynamics between participants (*i.e.,* within a walking group, group dynamics) and how they are affected by introducing a cognitive component to a PA intervention. This could be done, for instance, by assessing group cohesion or social identity formation. Additionally, observational methods, qualitative diaries, or more elaborate qualitative interviews focusing on the group dynamics, could provide deeper insights into participants’ experiences. Understanding these dynamics is essential for optimizing group-based interventions and ensuring that added components, such as cognitive tasks, enhance rather than hinder the social and psychological benefits of PA participation. Furthermore, to more clearly attribute potential psychosocial effects to PA or cognitively enriched PA, future studies should consider including a control group engaged in social activity only, either instead of or in addition to a passive control group.

Moreover, a more intensive data collection procedure, such as ecological momentary assessments (EMA), could provide more nuanced insights into how psychosocial well-being fluctuates in response to the intervention in real-time, rather than relying solely on pre-, mid-, and post-intervention measures.

## Conclusions

The group-based walking programs with and without cognitive enrichment did not elicit a significant change in depressive symptoms, positive well-being, loneliness, or social support. Nevertheless, about half of the participants of the walking program without enrichment and 40% of the participants of the cognitively enriched walking program did perceive a positive change in their psychosocial well-being. Data from the focus groups suggest that group walking programs (whether enriched or not) hold potential to establish new social connections, which can be long-lasting, and can have different functions for participants. Given the current demographic trends, this study offers important insights into the potential of group walking programs to contribute to promoting healthy aging.

## Supplemental Information

10.7717/peerj.20569/supp-1 Supplemental Information 1 Manual for coaches of WALK+ condition

10.7717/peerj.20569/supp-2 Supplemental Information 2 Semi-structured interview guide This was used for the focus groups

10.7717/peerj.20569/supp-3 Supplemental Information 3 Formula for the linear mixed model

10.7717/peerj.20569/supp-4 Supplemental Information 4 Example of a practice card (here “list learning”) as provided to the participants of WALK+

10.7717/peerj.20569/supp-5 Supplemental Information 5 Intervention effects on psychosocial well-being - results of the crude linear mixed models BDI-II: Beck Depression Inventory-II; WEMWBS: Warwick Edinburgh Mental Wellbeing Scale; SSL12-I: Social Support List-Interaction

10.7717/peerj.20569/supp-6 Supplemental Information 6 Main effects of time and condition

10.7717/peerj.20569/supp-7 Supplemental Information 7 The effects for the covariates

10.7717/peerj.20569/supp-8 Supplemental Information 8 Trial protocol

10.7717/peerj.20569/supp-9 Supplemental Information 9 CONSORT Checklist

## Additional Information and Declarations

### Data Availability

The following information was supplied regarding data availability: The datasets generated during and analyzed during the current study (restricted access, made available upon request), as well as the R script (open access), are available in the KU Leuven Research Data Repository: Hotterbeex, Pauline; van Uffelen, Jannique; Latomme, Julie; Beeckman, Melanie; Van Puyenbroeck, Stef; Chastin, Sebastien; Cardon, Greet, 2025, “Replication Data for: Walking Towards Psychosocial Well-being? Unveiling Psychosocial Impacts of a Group-Based Walking Program With and Without Cognitive Enrichment in Older Adults—A Mixed-Methods Randomized Controlled Trial”, https://doi.org/10.48804/KGWTR8, KU Leuven RDR, V1. The qualitative data is not shared as they contain identifiable information. Processing the request is done by the Research Data Repository (RDR) team of our university (rdr@kuleuven.be). The final decision on whether or not to share the data is made by the Data Protection Officer, Laurens Vangeel (dpo@kuleuven.be). To request access to the data (this is managed by a university-wide service): 1 - Upon submitting a request via the “Request Access” button, RDR reviewer/admin will be informed of the request. 2 - The RDR reviewer/admin will review the pre-defined, non-scientific, terms and forward the request to the responsible researcher. 3 - The responsible researcher will assess the pre-defined scientific terms and inform the RDR reviewer/admin of the decision, incl. reasoning. a. Positive evaluation: proceed to step 4 b. Negative evaluation with justified reasons: the request will be denied and requester will be informed. c. Negative evaluation without justified reasons: RDR reviewer/admin will refer the procedure to KU Leuven’s Data Protection Officer (DPO). 4 - When the request is deemed valid, the RDR reviewer/admin will send the tailored DTA to the requesting party, who will need to fill in their details in the DTA. The requesting party should present the DTA to an authorized person at their institute to ensure compliance with local regulations. 5 - After a final check by the RDR reviewer/admin, the DTA will be transferred to KU Leuven’s DPO for signing by the managing director. The requesting party will be asked to sign in the same PDF document. 6 - Access will be granted to the agreed upon (part of the) dataset.

### Clinical Trial Registration

The following information was supplied regarding Clinical Trial registration: NCT05500183.
