# Supplementary material for: Walking towards psychosocial well-being? Unveiling psychosocial impacts of a group-based walking program with and without cognitive enrichment in older adults—a mixed-methods randomized controlled trial
Source: PeerJ. 2026 Jan 22;14:e20569. doi: 10.7717/peerj.20569 (PMC12832057; doi:10.7717/peerj.20569)
Supplement: Supplemental Information 1 [file peerj-14-20569-s001.pdf]

Example of the description of a cognitive task (here “list learning”) as provided to the WALK+ coaches in the intervention manual.

LIST LEARNING
Memory

Goal

To memorize and retrieve lists. Trying to remember these lists for a period of time.

Explanation

During this task, participants have to practice memorizing lists, to be able to retrieve these at the end of the walk. The coach announces the elements one by one.

Framing (why)

This cognitive task aims to train the memory, more specifically the long-term memory.

Basic building blocks (mandatory)

1. Memorizing the lists
2. Leave enough time between the memorizing and retrieving the lists
3. Individually retrieving the lists (on paper or verbally)

Differentiation in difficulty

- The memorizing part can be done in pairs (easier because they can help each other) or alone
- When participants have difficulties remembering the lists, you can help them by providing memory techniques (e.g. try to make a word with the first letter of each word you have to memorize)
- The time between memorizing and retrieving the lists can be longer (more difficult) or shorter (easier)
- The longer the list, the more difficult

Variation content

- You can adapt the lists to be memorized to the season or interests of the participants
- Examples are: ingredients for a certain dish, emergency numbers, a poem (line by line), street names you pass

Important considerations

Make sure to leave enough time between announcing the different elements to remember, so participants have enough time to memorize each element.

Examples of lists

Recipe: Pesto

- Basil
- Parmesan cheese
- Olive oil

- Pepper
- Salt
- Pine nuts

A poem: One Art by Elizabeth Bishop

The art of losing isn't hard to master;
so many things seem filled with the intent
to be lost that their loss is no disaster.

Lose something every day. Accept the fluster
of lost door keys, the hour badly spent.
The art of losing isn't hard to master.

Then practice losing farther, losing faster:
places, and names, and where it was you meant
to travel. None of these will bring disaster.

I lost my mother's watch. And look! my last, or
next-to-last, of three loved houses went.
The art of losing isn't hard to master.

I lost two cities, lovely ones. And, vaster,
some realms I owned, two rivers, a continent.
I miss them, but it wasn't a disaster.

—Even losing you (the joking voice, a gesture
I love) I shan't have lied. It's evident
the art of losing's not too hard to master
though it may look like (Write it!) like disaster.
