# Supplementary material for: Walking towards psychosocial well-being? Unveiling psychosocial impacts of a group-based walking program with and without cognitive enrichment in older adults—a mixed-methods randomized controlled trial
Source: PeerJ. 2026 Jan 22;14:e20569. doi: 10.7717/peerj.20569 (PMC12832057; doi:10.7717/peerj.20569)
Supplement: Supplemental Information 2 — This was used for the focus groups [file peerj-14-20569-s002.pdf]

**Supplemental File S4**. Semi-structured interview guide. The questions in bold were used for this paper.

<table>
<tr><td></td><td>Participants</td></tr>
<tr><td>Opening Question</td><td>Could you briefly tell us your name, and in one sentence, your expectations for the program?</td></tr>
<tr><td>Introductory Question</td><td>Can you indicate in one word what describes your experience with the program?</td></tr>
<tr><td>Transition Question</td><td>Can you describe your general experiences with the program?</td></tr>
<tr><td>Key Questions</td><td>What did you find to be the good aspects of the program?<br>Specifically address: social aspect<br>What did you find to be the less good aspects of the program?<br>Specifically address: difficulty of exercises*, walking intensity, unsupervised sessions*<br>How did you experience the interaction with the coach?<br>What changes did you experience during the period of the program? This can relate to physical, cognitive, psychological, or social aspects. (15 minutes)<br>• If changes were experienced: When did you first notice them? What do you think caused these changes?<br>• If no changes were experienced: What do you think might be the reason for that?<br>What would be reasons to continue participating or to stop participating in the program?<br>In what way could the program be improved?*</td></tr>
<tr><td>Concluding questions</td><td>If we had all the resources in the world, what would the ideal program look like for you?*<br>What is, for you, the most important thing said today? (If you could choose only one thing)<br>Would you like to add anything to what has been said about your experiences with the program?</td></tr>
</table>

*only for WALK+; ^only for WALK-only
