# Supplementary material for: Walking towards psychosocial well-being? Unveiling psychosocial impacts of a group-based walking program with and without cognitive enrichment in older adults—a mixed-methods randomized controlled trial
Source: PeerJ. 2026 Jan 22;14:e20569. doi: 10.7717/peerj.20569 (PMC12832057; doi:10.7717/peerj.20569)
Supplement: Supplemental Information 3 [file peerj-14-20569-s003.pdf]

Supplementary File S5. Formula for the linear mixed model

$Y_{ij} = \beta_0 + \beta_1 * Time_{ij} + \beta_2 * Condition_i + \beta_3 * (Time_{ij} \times Condition_i) + \beta_4 * Age_i + \beta_5 * Sex_i + \beta_6 * CivilStatus_i + \beta_7 * MVPA_i + \upsilon_{0i} + \varepsilon_{ij}$

In this model:

- β_0: the fixed intercept
- $\beta_1 * Time_{ij}$ represents within-person change over the time points (baseline, 3 months, 6 months)
- $\beta_2 * Condition_i$ represents the between-person effect of condition (WALK+, WALK-only or CONT)
- $\beta_3 * (Time_{ij} \times Condition_i)$ represents the interaction term whether the change in the outcome over time differs between groups
- β_4-β_7: between-person effects of covariates
- υ_{0i} is the random intercept for each participant (i.e. the random deviation for each person from the grand mean) to account for repeated measures
- ε_{ij}: within-person residual error

This corresponds to the code in the R script that is made available on the KU Leuven Research Data Repository (https://rdr.kuleuven.be/privateurl.xhtml?token=e5ddf736-b7c5-46fa-a04d-ffa8347238e7):

*lmer(outcome ~ 1 + Time_Fact*Condition_Fact + Age_Baseline + Sex_MM1 + Civil_Status_MM1 + Baseline_MVPA + (1|ID))*
