# Supplementary figures and images for: Walking towards psychosocial well-being? Unveiling psychosocial impacts of a group-based walking program with and without cognitive enrichment in older adults—a mixed-methods randomized controlled trial
Source: PeerJ. 2026 Jan 22;14:e20569. doi: 10.7717/peerj.20569 (PMC12832057; doi:10.7717/peerj.20569)

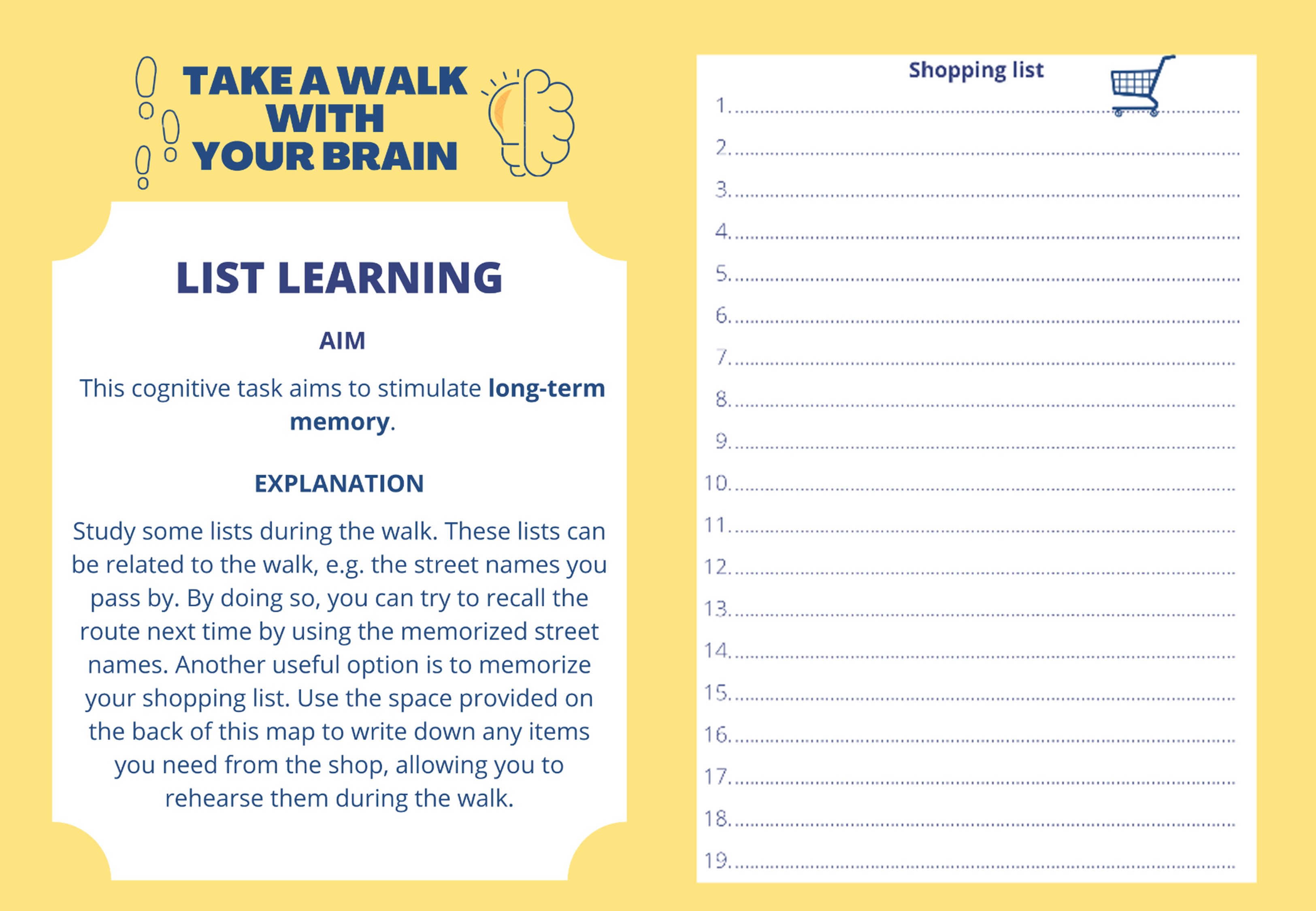

Supplement: Supplemental Information 4 [file peerj-14-20569-s004.jpg]
