# Supplementary material for: Walking towards psychosocial well-being? Unveiling psychosocial impacts of a group-based walking program with and without cognitive enrichment in older adults—a mixed-methods randomized controlled trial
Source: PeerJ. 2026 Jan 22;14:e20569. doi: 10.7717/peerj.20569 (PMC12832057; doi:10.7717/peerj.20569)
Supplement: Supplemental Information 5 — BDI-II: Beck Depression Inventory-II; WEMWBS: Warwick Edinburgh Mental Wellbeing Scale; SSL12-I: Social Support List-Interaction [file peerj-14-20569-s005.pdf]

**Supplementary Table 1**

**Intervention effects on psychosocial well-being (linear mixed models - crude)**

| | **WALK+ vs. WALK-only** | | | | **WALK+ vs. CONT** | | | | **WALK-only vs. CONT** | | | |
|---|---|---|---|---|---|---|---|---|---|---|---|---|
| | **β** | **df** | **95%CI** | **p** | **β** | **df** | **95%CI** | **p** | **β** | **df** | **95%CI** | **p** |
| **Depressive symptoms** | | | | | | | | | | | | |
| (BDI-II) n=148 | | | | | | | | | | | | |
| Pre vs. 3m | 0.21 | 264 | -1.49;1.91 | 0.81 | -0.03 | 266 | -1.78;1.71 | 0.97 | -0.25 | 265 | -1.99;1.50 | 0.78 |
| Pre vs. 6m | -0.26 | 264 | -1.97;1.45 | 0.77 | -1.10 | 267 | -2.87;0.66 | 0.22 | -0.85 | 265 | -2.62;0.92 | 0.35 |
| **Positive well-being** | | | | | | | | | | | | |
| (WEMWBS) n=148 | | | | | | | | | | | | |
| Pre vs. 3m | 0.71 | 257 | -1.24;2.65 | 0.45 | -0.56 | 255 | -2.59;1.47 | 0.59 | -1.22 | 259 | -3.24;0.80 | 0.24 |
| Pre vs. 6m | 1.10 | 257 | -0.87;3.06 | 0.33 | 1.11 | 254 | -0.94;3.15 | 0.29 | 0.01 | 258 | -2.03;2.04 | 0.99 |
| **Loneliness** | | | | | | | | | | | | |
| (De Jong-Gierveld) n=145 | | | | | | | | | | | | |
| Pre vs. 3m | 0.53 | 251 | -0.32;1.39 | 0.22 | 0.16 | 253 | -0.74;1.05 | 0.73 | -0.38 | 252 | -1.27;0.51 | 0.40 |
| Pre vs. 6m | -0.18 | 251 | -1.04;0.67 | 0.68 | -0.49 | 252 | -1.38;0.40 | 0.28 | -0.31 | 251 | -1.20;0.59 | 0.50 |
| **Social support** | | | | | | | | | | | | |
| (SSL12-I) n=148 | | | | | | | | | | | | |
| Pre vs. 3m | -0.25 | 260 | -2.05;1.54 | 0.78 | 0.11 | 263 | -1.76;1.97 | 0.91 | 0.36 | 261 | -1.51;2.23 | 0.71 |
| Pre vs. 6m | 0.74 | 260 | -1.08;2.55 | 0.43 | 0.37 | 263 | -1.51;2.25 | 0.70 | -0.37 | 261 | -2.25;1.52 | 0.70 |

BDI-II: Beck Depression Inventory-II; WEMWBS: Warwick Edinburgh Mental Wellbeing Scale; SSL12-I: Social Support List-Interaction
