# Supplementary material for: Walking towards psychosocial well-being? Unveiling psychosocial impacts of a group-based walking program with and without cognitive enrichment in older adults—a mixed-methods randomized controlled trial
Source: PeerJ. 2026 Jan 22;14:e20569. doi: 10.7717/peerj.20569 (PMC12832057; doi:10.7717/peerj.20569)
Supplement: Supplemental Information 6 [file peerj-14-20569-s006.pdf]

Main effects of time for WALK+						
	Pre – 3m			Pre – 6m		
	**β**	**95%CI**	**p**	**β**	**95%CI**	**p**
**Depressive symptoms**	-0.13	-1.35;1.10	0.84	0.51	-0.72;1.74	0.42
**Positive well-being**	-0.55	-1.93;0.83	0.43	-0.73	-2.13;0.66	0.30
**Loneliness**	0.07	-0.5;0.68	0.83	0.45	-0.17;1.07	0.15
**Social support**	-0.09	-1.36;1.19	0.90	-0.51	-1.80;0.77	0.43

Main effects of condition									
	WALK+ vs. CONT			WALK+ vs. WALK-only			WALK-only vs. CONT		
	**β**	**95%CI**	**p**	**β**	**95%CI**	**p**	**β**	**95%CI**	**p**
**Depressive symptoms**	1.59	-0.31;3.48	0.10	0.55	-1.36;2.45	0.57	1.04	-0.87;2.96	0.29
**Positive well-being**	-2.06	-4.68;0.57	0.13	-2.29	-4.92;0.34	0.09	0.24	-2.41;2.89	0.86
**Loneliness**	0.64	-0.71;2.00	0.35	-0.37	-1.72;0.98	0.59	1.01	-0.36;2.38	0.15
**Social support**	-1.95	-4.04;0.15	0.07	-0.34	-2.43;1.75	0.75	-1.60	-3.72;0.51	0.14
