# Supplementary material for: Walking towards psychosocial well-being? Unveiling psychosocial impacts of a group-based walking program with and without cognitive enrichment in older adults—a mixed-methods randomized controlled trial
Source: PeerJ. 2026 Jan 22;14:e20569. doi: 10.7717/peerj.20569 (PMC12832057; doi:10.7717/peerj.20569)
Supplement: Supplemental Information 7 [file peerj-14-20569-s007.pdf]

Supplementary File S7. Effects of the covariates on psychosocial well-being outcomes

		Depressive symptoms	
	**β**	**95% CI**	**p**
**Age**	-0.13	-0.29;0.04	0.13
**Sex**	-0.09	-1.68;1.49	0.91
**Civil status**	-0.57	-1.37;0.24	0.16
**Baseline PA**	-0.01	-0.03;0.01	0.22
		**Positive well-being**	
	**β**	**95% CI**	**p**
**Age**	0.08	-0.16;0.32	0.53
**Sex**	0.64	-1.65;2.93	0.58
**Civil status**	0.28	-0.88;1.43	0.64
**Baseline PA**	0.03	-0.002;0.06	0.07
		**Social support**	
	**β**	**95% CI**	**p**
**Age**	-0.02	-0.20;0.17	0.86
**Sex**	2.91*	1.14;4.68	0.001
**Civil status**	-0.62	-1.51;0.27	0.17
**Baseline PA**	-0.002	-0.02;0.02	0.84
		**De Jong-Gierveld Loneliness**	
	**β**	**95% CI**	**p**
**Age**	-0.08	-0.21;0.04	0.20
**Sex**	-0.85	-2.05;0.35	0.16
**Civil status**	-0.46	-1.06;0.15	0.14
**Baseline PA**	-0.01	-0.02;0.01	0.49
