# Supplementary material for: Walking towards psychosocial well-being? Unveiling psychosocial impacts of a group-based walking program with and without cognitive enrichment in older adults—a mixed-methods randomized controlled trial
Source: PeerJ. 2026 Jan 22;14:e20569. doi: 10.7717/peerj.20569 (PMC12832057; doi:10.7717/peerj.20569)
Supplement: Supplemental Information 8 [file peerj-14-20569-s008.pdf]

Protocol and statistical analysis plan

Real-life cognitively enriched walking program for people aged ≥65 years

National Clinical Trial (NCT) Identified Number: NCT05500183

Principal Investigators: Prof. Greet Cardon (Ghent University) and Prof. Jannique van Uffelen (KU Leuven)

Funded by: Research Foundation Flanders (FWO)

Grant number: G009819N

Last updated: 30/05/2024

1. Introduction

Dementia currently affects more than 47 million people worldwide, its prevalence is forecasted to triple by 2050, and it has been reported to be one of the most costly disorders in Belgium.

There is good scientific evidence that the cognitive impairments associated with the development of dementia can be lessened or even reversed thanks to the plasticity of the brain (rewiring). Recent research has shown that physical activity combined with performing cognitively challenging tasks is a very potent way to induce this rewiring of the brain, which can enable people to improve their cognitive functions. Yet, so far, these studies are mainly limited to controlled laboratory conditions. Our team developed a real-life cognitively enriched walking program, with input from experts and end-users.

In this study, we will examine the added value of enriching physical activity (walking) with cognitive exercises in improving cognition of older adults by conducting a six-month community-based randomized controlled trial. We will also examine the longer term effectiveness in a follow-up measurement visit six months after the program. We will focus on the following outcomes: cognitive functioning (i.e., objective, subjective and cognitive activity), psychosocial wellbeing (i.e., loneliness, social support, depressive symptomatology, positive wellbeing and expectations regarding aging), physical activity (i.e. both objective and subjective) and general health.

2. Study design

To evaluate the effects of the cognitively enriched walking program on cognitive functioning (primary outcome), physical activity and psychosocial wellbeing (secondary outcomes), a randomized controlled trial (RCT) will be conducted. The intervention period will be 6 months, with measurements conducted at baseline, 3 months (mid-intervention), 6 months (post-intervention) and 12 months (follow-up). The interventions and measurements will take place at 2 university campuses in Belgium (Ghent and Leuven).

Participants will be allocated to one of the following intervention arms with a 1:1:1 allocation ratio: (1) cognitively enriched walking program, (2) walking program without enrichment, (3) passive control group. An investigator not involved in running the trial will generate the randomization sequence stratified by location (Ghent/Leuven) and availability (2 scheduling options for each location) using a statistical software program. Allocation will be disclosed to the researchers and participants only after completion of baseline measurements, ensuring double-blind data collection at baseline.

Furthermore, a process evaluation will be embedded in the RCT, using a mixed-methods design, and thus comprises questionnaires and focus group interviews. Questionnaires will be completed mid-intervention and post-intervention, focus groups will be conducted post-intervention.

3. Participants

3.1. Recruitment

Participants for this study will be recruited by convenience and snowball sampling. Flyers with basic information on the study, the researcher's contact information (i.e. e-mail address and phone number), and name of the study's website will be distributed through different channels. Examples of these channels are local service centres, community health centres, stakeholders and organisations for older adults (e.g. OKRA, Vief, S-plus, Vlaamse Ouderenraad), social media and mail to people who previously showed interest in this program and provided their contact details (e.g. people that were not selected for participation in the pilot study but agreed to be kept informed about this study, people reached through an information day (Care4Brain) of the Leuven Brain Institute). On the website, we will share a brief summary of what is expected of study participants. Furthermore, our in- and exclusion criteria as well as our contact information will be listed on the website. It will also be stated that during an initial phone call, the in- and exclusion criteria will be discussed and that only with participants meeting these criteria, an appointment will be made for the baseline measurement. We will also try to specifically reach neighbourhoods of people with lower socioeconomic status, because these people are often less physically active. We will do this by distributing our flyers in community (health) centres in person, to have

a first personal contact with our potential participants. Furthermore, an advertisement will be placed in a local newspaper and/or on Facebook and we will organize live information sessions for all those interested. To ensure the handed information is understandable for everyone, we will use no specialist terms on our flyers or website.

We will also recruit coaches, who will supervise the sessions. These coaches will be hired, and thus paid, for their contribution to this project. To recruit them, will contact Wandelsport Vlaanderen saying that we are looking for certified walking coaches for this particular project, including a flyer that can be distributed. They will then contact the suited walking initiators and let us know who is interested in this project. By certified, we mean that the coaches have completed the training 'walking initiator' of Wandelsport Vlaanderen and Vlaamse Trainersschool.

3.2. Inclusion and exclusion criteria

We will include male and female volunteers aged 65 years and older. The age of 65 years is a well-accepted cut-off as well as the age at which most people can retire in Belgium. This means that people of this age group will be more likely to have enough free time to participate in this study. Participants have to be community-dwelling.

Because we aim to investigate the effects of the cognitively enriched walking program on cognitive function of healthy older adults, potential participants will be excluded if (they):

(1) Have a neurodegenerative disorder (i.e. Alzheimer's disease, Parkinson's disease, Multiple Sclerosis,…);
(2) Are diagnosed with mild cognitive impairment (MCI);
(3) Are diagnosed with a psychiatric disorder (e.g. schizophrenia, bipolar disorder, borderline personality disorder,…);
(4) Are currently having a depressive episode;
(5) Have had a serious brain injury in the past year, or before and still experience consequences (i.e. traumatic brain injury, stroke, brain haemorrhage);
(6) Have a history of, or current, addiction to drugs or excessive alcohol abuse;
(7) Are not able to walk approximately three kilometres in one hour (at an average walking pace);
(8) Make use of a walking aid (i.e. a cane, a (rollator) walker, or a wheelchair);
(9) One or both of their parents were diagnosed with juvenile dementia;
(10) Know in advance that they will not be will not be present for one or more periods of longer than one month (e.g. because of a long holiday).

3.3. Screening

Since the focus of this study is evaluating this program in cognitively and physically healthy older adults and cognitive functioning is our primary outcome, we decided to add two objective criteria to exclude participants that are more likely to have cognitive impairment. Firstly, participants with a score of less than 23 on the Montreal Cognitive Assessment tool (MoCA) will be excluded, as this might indicate (the beginning of) mild cognitive impairment (Carson et al., 2018). Additionally, we will exclude participants with a score of 20 and above (i.e. moderate to severe depression symptoms (Balsamo et al., 2018) on the Beck Depression Inventory (BDI-II), as depression can impact cognitive functioning (Köhler et al., 2010). Whenever participants are excluded because of a MoCA score below or BDI-II score above the cut-off, the participant will be informed about the deviant score by the either the PhD student (who has a MSc in Psychology and did an eight-month internship at a geriatric's department) or the postdoctoral researcher (who has a MSc and PhD in Psychology, took a course in psychogeriatrics, worked at a geriatric's department and works part-time as a primary care psychologist). Furthermore, the person will receive a letter that they can bring at their next appointment with their general practitioner. The letter will (1) contain the description of the test and the score obtained and an explanation that there are multiple possible reasons for such a score, and that only one of them is (mild) cognitive impairment or depressive symptomatology and (2) indicate that, in case the general practitioner thinks this is necessary, an appointment can be made with a memory clinic or psychologist.

4. Interventions

As mentioned above, there will be three intervention arms: 1) a cognitively enriched walking program (Physical Activity (PA)+Cognitive Activity (CA)), 2) a walking program without cognitive enrichment (Physical Activity (PA) only) or 3) a passive control group (CG). The passive CG will receive no intervention program. Both the PA+CA and the PA program are group-based and will have a duration of six months (i.e. 24 weeks), with a frequency of two group sessions a week. All group sessions will last for approximately one hour and take place in groups of twelve or thirteen people supervised by a certified coach. Participants in both intervention groups (PA+CA and PA) will be encouraged to do one individual session every week, as well.

The PA+CA group will perform cognitive exercises while walking. One cognitively enriched walking session can be divided in three parts: (1) 5-10 minutes of brisk walking, (2) approximately 30-40 minutes of cognitive exercises while walking and (3) 5-10 minutes of cooling down (i.e. time for small talk or rest and relaxation). Cognitive exercises are exercises that are cognitively challenging and focus on multiple cognitive functions (i.e. attention, memory, executive functions, language, creative thinking) and can be performed while walking. Some examples are: planning a route, solving riddles, noticing, word fluency, bingo (i.e. noticing things along the walk),… These cognitive exercises have been developed in agreement with experts and the target population (Marent et al., 2021). Every cognitively enriched walking session, two to three cognitive exercises will be explained by the coach and performed by the participants. To this end, the coach receives a standardized manual with explanations of the different cognitive exercises. Furthermore, the coach will make sure that participants keep moving while performing the cognitive exercises. For the individual sessions, participants of the PA+CA group will receive practice cards with simplified instructions for all cognitive exercises that can be performed alone, with their partner, friends, or even grandchildren. Participants will be asked to register the number of individual cognitively enriched walks they perform in a personal log-book.

The PA group will walk for approximately the same amount of time and distance as the PA+CA group, but will not perform cognitive exercises. The coach will supervise and guide the group walk. These participants will also be asked to register the amount of individual walks they perform in a personal log-book.

The passive CG will not receive an intervention. Participants will be informed that they are assigned to the control group.

Participants will receive a letter at home with information about the intervention group they are allocated to (i.e. when will the sessions take place, where will they take place, contact information of the coach). Participants will be asked to be present for as many sessions as possible, but given the real-life context of this study, we will have the coaches track the attendance (in both the PA+CA and PA groups) so we can account for this in the analyses. The control group will receive information about why the use of a control group is important in intervention studies to keep them motivated to attend the following measurement sessions.

Debriefing

All participants will be debriefed after the end of the study. This will be done by providing them with a report that will include information about the aim of this study and the different intervention conditions, the results of the study and their own performance during the study. It is practically and financially not feasible to organize the cognitively enriched walking intervention again for six months for the PA group and CG. Therefore, all participants will be given access to all intervention materials (e.g. the manual for the coaches) so they can try the cognitively enriched walking by themselves.

5. Outcome measures

5.1. Effectiveness

The main outcome of this study is cognitive functioning. We chose several outcome measures to assess different aspects of **cognitive functioning**.

- *Cambridge Neuropsychological Test Automated Battery (CANTAB) (Bento-Torres et al., 2017; Sahakian & Owen, 1992; Zygouris & Tsolaki, 2015):* objective cognitive functioning is assessed with CANTAB.

Different tests can be configured in a test battery, based on the focus of the study. The tests are non-verbal and culturally independent and make use of touchscreen technology. The CANTAB has been shown sensitive to changes in cognitive performance. Six different tests were selected to obtain a comprehensive view of one's objective cognitive functioning. The selected tests assess psychomotor speed/attention (i.e. sustained attention), memory (i.e. visual episodic attention and short term visual memory) and executive functioning (i.e. working memory and strategy). This is the primary endpoint of the study

- *Cognitive and Leisure Activities Scale (CLAS)(Galvin et al., 2021):* this is a measure of the type and frequency of cognitive activities. It includes sixteen types of cognitive activities and is self-administered. Even though it is difficult to establish the validity of this new questionnaire since there is no gold standard to measure cognitive activity, it is suggested that this questionnaire has a good content validity. This will be used as a covariate in the analysis.

- *Cognitive Failures Questionnaire (CFQ) (Broadbent et al., 1982; Ponds et al., 2006):* this self-administered questionnaire assesses subjective cognitive functioning. Participants are asked about minor everyday slips or errors. It is not a replacement for testing objective cognitive functioning, but reflects the experience of the frequency of everyday slips of the participant.

Psychosocial wellbeing

- *Beck Depression Inventory (BDI-II) (Balsamo et al., 2018):* this self-administered questionnaire is part of our screening procedure, but will be administered during all measuring points to assess the evolution of depressive symptomatology during and after the intervention. There is evidence supporting the construct, convergent and discriminant validity of the BDI-II.

- *Warwick-Edinburgh Mental Wellbeing Scale (WEMWBS) (Tennant et al., 2007):* this is a measure of wellbeing, focussing on positive mental health. This measure is suggested to be able to assess improvements in mental health wellbeing. This scale has robust psychometric properties.

- *De Jong Gierveld short scale for loneliness (De Jong Gierveld & Van Tilburg, 2008; De Jong Gierveld & Van Tilburg, 2010):* this is a six-item questionnaire with a threefold application. It assesses overall loneliness, as well as emotional loneliness and social loneliness. Good validity and reliability has been reported for this questionnaire.

- *Social Support List – Interaction (SSL 12 – I) (Kempen & van Eijk, 1995; van Eijk et al., 1994):* this is a twelve-item questionnaire with satisfactory psychometric properties that was especially developed for use in older adults. It assesses three dimensions of social support: daily support, support in case of problems and appreciation.

- *Expectations Regarding Aging (ERA-12) (Sarkisian et al., 2005):* this twelve-item questionnaire assesses older adult's expectations regarding aging. It provides us with a general score as well as a separate score for the expectations regarding cognitive, mental and physical health. Acceptable reliability and validity to estimate older adults' expectations regarding aging has been established.

Physical activity

- *International Physical Activity Questionnaire – Short Form (IPAQ-SF) (Craig et al., 2003):* this is an extensively used self-report measure for physical activity during the past seven days. It provides us with a categorical score of physical activity, distinguishing between low, moderate and high levels of physical activity.

- *Actigraph accelerometer at hip (Barnett et al., 2016):* this is an objective measure of physical activity level, frequently used in physical activity studies with older adults. Participants will be asked to wear this accelerometer during seven days (day and night). They will be asked to register when and why they did not wear the device in a personal diary provided by the researchers. The duration spent in moderate-to-vigorous PA (based on the cutpoints of Barnett et all. (2016)) will be used as an outcome in this study.

General health

- *The Patient-Reported Outcomes Measurement Information System (PROMIS) eight-item short form for General Health (Pellicciari et al., 2021)*: this is a self-report measure for global self-rated health with acceptable psychometric properties. This questionnaire provides us with a total score as well as distinct scores for mental and physical health.

- *The Patient-Reported Outcomes Measurement Information System (PROMIS) four-item short form for Sleep Disturbances (Buysse et al., 2010; Terwee et al., 2014)*: this self-report measure with good psychometric properties assesses quality of sleep and prevalence of sleep difficulties.

5.2. Process evaluation

To gain a meaningful insight into the relation between different components of the new program and the outcomes, we will include a process evaluation. For this, we will use both quantitative as well as qualitative methods. Participants of both the PA+CA as the PA groups will be asked to complete a process evaluation questionnaire after three and six months of the intervention. This will include questions on fidelity, delivered dose (completeness), received dose (exposure, satisfaction), participation rate, acceptability of the intervention, group dynamics, environmental context,…

As the use of both qualitative and quantitative data provides the strongest evidence for process evaluation, we will collect both types of data in this study. Process evaluation tools were developed based on the framework of Saunders et al. (2005), which recommends to evaluate six key elements in a process evaluation: (1) fidelity (quality of the implementation of the intervention), (2) dose delivered (completeness, amount of components provided by the coaches), (3a) dose received – exposure (extent to which participants actively engage with materials), (3b) dose received – satisfaction (participant satisfaction), (4) reach (participation rate), (5) recruitment (procedures used) and (6) context (aspects of the environment that may influence implementation or study outcomes). In the table below, the data sources and data collection tools are summarized.

		**Data sources**	**Tools**	**Timing/frequency**
1	Fidelity (quality)	Participants and coaches	• Questionnaires and focus groups	• Both post intervention
2	Dose delivered (completeness)	Coaches	• Questionnaire and focus groups	• Both post intervention
3a	Dose received (exposure)	Participants	• Questionnaire and focus groups<br>• Registration form	• Post intervention<br>• During the intervention
3b	Dose received (satisfaction)	Participants	• Questionnaire and focus groups	• Post intervention
4	Reach	Coaches	• Attendance list	• During the intervention
5	Recruitment	Main researcher	• Documentation of all recruitment activities	• During recruitment phase

6	Context	Participants and coaches	• Questionnaire and focus groups	• Post intervention

Table 1. Process evaluation elements, data sources, tools and timing/frequency

Questionnaires

Questionnaires consist of both closed (Likert scales) and open-ended questions. Separate questionnaires have been drafted for participants and coaches of both intervention groups (i.e., four questionnaires in total: coaches PA only, coaches PA+CA, participants PA only, participants PA+CA). Participants and coaches will receive a link to an online questionnaire (UGent Qualtrics). Coaches are asked to identify themselves by indicating in which city they are guiding a group, and on which days. Furthermore, coaches are also asked to provide sociodemographic information: gender, age and educational degree. The questionnaires for the coaches are attached to this proposal as additional files 1 and 2.

Focus groups

All participants of the PA only and PA+CA groups will be invited to participate in focus groups. In total, there are four groups performing the PA only program (two in Leuven and two in Ghent) and four groups performing the PA+CA program (two in Leuven and two in Ghent). We will organize eight focus groups for participants, one for each group. Each focus group will consist of 8-13 participants.

There are four coaches guiding the PA only groups and four coaches guiding the PA+CA groups. Therefore, we opted to organize one focus group for the coaches of the PA only groups (from both Ghent and Leuven) and one focus group for the coaches of the PA+CA groups (from both Ghent and Leuven). Each focus group for coaches will thus consist of 4 participants.

Every focus group will take around 60-90 minutes. Focus groups for participants will be organized on campus (in Ghent or Leuven, depending on where participants were part of an intervention group). Focus groups for coaches will be organized on a KU Leuven campus in Brussels (because coaches from Ghent and Leuven will participate in the same focus group). For all focus groups, a semi-structured interview guide will be used. In total, four separate interview guides are prepared: 1) coaches of the PA only groups, 2) coaches of the PA+CA groups, 3) participants of the PA only groups, 4) participants of the PA+CA groups. The PhD student will be moderator of all focus groups, the postdoctoral researcher and/or a master student will be observer. All focus groups will be audio-recorded, and the observer will take notes during the focus groups. The interview guides are attached as additional file 3, 4, 5 and 6.

5.3. Procedures

Screening

A telephone call will be arranged with people interested in participating in this study. During this phone call with one of the researchers, the above-mentioned inclusion and exclusion criteria will be checked. Potential participants that meet the inclusion criteria will be invited for a baseline measurement visit. They will also receive a digital version of the information letter and informed consent, so they can read this before signing it during the baseline visit.

Baseline visit

First, the informed consent will be signed. At this moment, participants can also ask questions at the researcher if they have any after reading the information letter. During the baseline visit, the MoCA and BDI-II will be administered as a secondary screening for possible cognitive difficulties and depressive symptomatology, respectively. As mentioned above, participants excluded from the study at this stage will be informed about the deviant score by the either the PhD student or the postdoctoral researcher. Furthermore, the person will receive a letter that they can bring at their next appointment with their general practitioner. The letter will (1) contain the description of the test and the score obtained and an explanation that there are multiple possible reasons for such a score, and that only one of them is (mild) cognitive impairment or depressive symptomatology and (2)

indicate that, in case the general practitioner thinks this is necessary, an appointment can be made with a memory clinic or psychologist.

Included participants will be asked to provide sociodemographic information (i.e. age, gender, length, weight, nationality, country of origin, educational level, marital status, longest held profession, and income) during this baseline visit. Furthermore, participants will be asked if there is any relevant medical information the coaches need to know to ensure safety of the waling sessions (i.e., so the coach can anticipate any possibly dangerous situations).

Participants will also be asked to complete the questionnaires regarding cognitive function, psychosocial wellbeing, physical activity and general health described above (see *outcomes*). Objective cognitive and physical activity will be measured at baseline as well by administering the CANTAB and wearing an accelerometer for seven consecutive days (day + night), respectively. Participants will be asked to return the accelerometer by delivering it to the office of one of the researchers or by returning it by post (pre-paid by the research team).

Three months visit

Three months after the start of the intervention, participants will be invited for a second visit. Again they will be asked to complete the questionnaires regarding cognitive function, psychosocial wellbeing, physical activity and general health. The CANTAB will be administered and they will be asked to wear the accelerometer for seven consecutive days. This measurement visit will take place during the intervention period for the PA+CA and PA groups, this will be taken into account in the analysis of the accelerometer data. Furthermore, a process evaluation questionnaire will be given to participants of the PA+CA and PA groups. This questionnaire can be completed at home and returned during the next (cognitively enriched) walking session. Accelerometers can be delivered to the office of one of the researchers or returned by post (pre-paid by the research team).

Six months visit

After the end of the intervention, participants will be invited for a third visit. The procedure is the same as that of the second visit. However, participants will not be taking part in an intervention while wearing the accelerometer. Participants will be asked to return the process evaluation questionnaires as well as the accelerometer, either by delivering it to the office of one of the researchers or returning it by post (pre-paid by the research team).

Follow up visit

Six months after the intervention end, so twelve months after the intervention start, there will be one last follow-up visit. The procedure is again the same as that of the second and third visit, except that participants will not have to complete a process evaluation questionnaire. Again, we will ask participants to return the accelerometers by delivering it to the office of one of the researchers or by returning it by post (pre-paid by the research team).

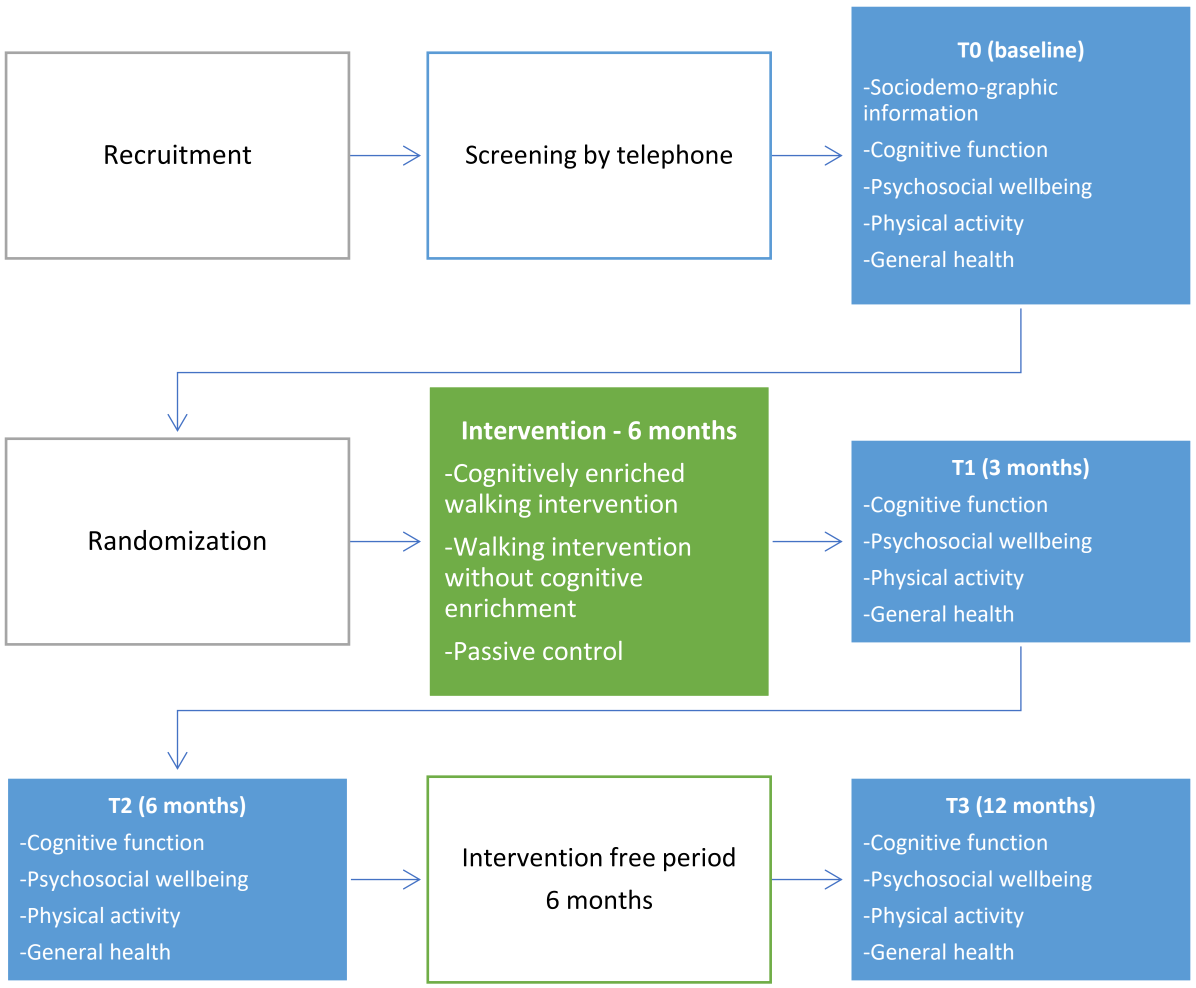

Figure 1. Procedure

6. Statistical analysis plan

6.1. Hypotheses

Primary endpoint:

We hypothesize that the cognitively enriched walking program will have more beneficial effects on cognitive functioning compared to the other conditions at 6 months and 12 months.

Secondary endpoints:

We hypothesize that both the cognitively enriched walking program and the walking program without cognitive enrichment will have more beneficial effects on moderate-to-vigorous PA levels compared to the control group.

We hypothesize that both the cognitively enriched walking program and the walking program without cognitive enrichment will have more beneficial effects on psychosocial wellbeing compared to the control group.

Process evaluation:

For this endpoint, we do not have a predefined hypothesis. This will thus be an exploratory analysis.

6.2. Sample size

An a priori power calculation was conducted in G*Power 3.1. With an estimated effect size of 0.30 (average effect size for comparison between PA+CA and control across 3 meta-analyses of Zhu et al. (2016), Gavelin et

al. (2021) and Gheysen et al. (2018)), α=0.05, power=0.80, a sample size of 124 would be sufficient to detect differences between the conditions. Assuming a 20% dropout rate, a total of 149 participants will be recruited.

6.3. Populations for analyses

The data set will be analysed according to the ‘intention to treat’ (taking into account everyone who was assigned to an intervention arm) and ‘per protocol’ (taking into account only those who received ≥75% of the intervention, and the control group) principle.

6.4. Statistical analyses

General

For descriptive statistics, categorical data will be presented as percentages and counts, continuous data will be presented as means with standard deviations or medians with interquartile range in case of non normally distributed data.

For inferential statistics, α will be set at 0.05, and 95% confidence intervals will be calculated. In case of multiple testing, a correction of the p-values will be used.

Covariates will be specified below and are selected based on theoretical knowledge.

Checks of assumptions will be performed, in case of non-normality, transformations of the variables or non-parametric tests will be used.

Primary outcome

For each cognitive test used, a selection of outcome measures will be used based on literature. Next, composite scores will be constructed for short-term memory (STM), long-term memory (LTM), executive functioning (EF) and processing speed (PS). The procedure for this is described below

The outcome measures, categorized by cognitive function:

**Short-term memory (STM)**	**Long-term memory (LTM)**	**Executive functioning (EF)**	**Processing speed (PS)**
VRM free recall distinct stimuli (VRMFRDS)	VRM delayed recognition total correct (VRMDRTC)	SWM strategy (SWMSX)	RTI reaction time (RTIFMDRT)
VRM immediate recognition total correct (VRMIRTC)		SWM between errors levels 4, 6, 8 and 12 (SWMBE46812)*	
PAL first attempt memory score (PALFAMS28)		OTS problems solved upon first choice (OTSPSFC)	
PAL total errors (PALTEA28)			
DMS percentage correct (DMSPCAD)			

*SWMBE46812 is calculated by summing SWMBE4, SWMBE6, SWMBE8, SWMBE12

STEP 1: Create z-scores based on the pooled mean (across conditions), separately for each time point

STEP 2: Reverse-score the z-scores for the following outcome measures:

PALTEA

SWMSX

SWMBE46812

RTIFMDRT

STEP 3: Calculate the composite scores using the following formulas

STM = (VRMDRDS + VRMIRTC + PALFAMS28 + PALTEA28 + DMSPCAD)/5

LTM = VRMDRTC

EF = (SWMSX + SWMBE46812 + OTSPSFC)/3

PS = RTIFMDRT

The outcomes are measured repeatedly at different time points, and are nested within individuals. This implies our data has a two-level structure, and this calls for an analysis technique taking into account the correlation between observations from the same person (Hoffman & Walters, 2022). Therefore, linear mixed models with random intercepts will be used, to take into account that each individual participant has a different starting point. Time (baseline, 3m, 6m and 12m) and condition (PA+CA, PA only, CG), and their interaction will be included as fixed effects.

Models with different levels of adjustment will be run: (1) crude model, (2) partially adjusted model including age, sex and education as covariates, and (3) fully adjusted model including age, sex, education, baseline PA and baseline CA as covariates. Model fit indices will be used to choose the models with the best fit.

In case of significant effects, post-hoc pairwise comparisons will be conducted to examine differences in the estimated marginal means of the outcome at T0, T1, T2 and T3 between 1) WALK+ vs. WALK-only and 2) WALK+ vs. CONT, but also the changes from T0 to T1, T0 to T2, T0 to T3, T1 to T2 and T2 to T3 for each group (WALK+, WALK-only and CONT). The p-values will be corrected for multiple testing.

The analysis will be conducted for the intention-to-treat and per protocol populations.

Secondary outcomes

For physical activity, the Choi et al. (2011) algorithm will be used to define (non-)wear time. Next, the cutpoints of Barnett et al. (2016) will be used to determine time spent in moderate-to-vigorous physical activity.

Other outcomes will be processed as indicated in their corresponding manuals.

The same analysis as for the primary outcome will be used. The only difference will be the covariates: for physical activity these will be age and sex, for psychosocial wellbeing these will be age, sex and civil status.

The analysis will be conducted for the intention-to-treat and per protocol populations.

Process evaluation

Quantitative data from the questionnaires will be handled in SPSS IBM Corp (2021). Descriptive statistics (frequencies, percentages, median and range or mean and standard deviations) of the responses to the questions will be generated.

Audio-recordings of the focus groups will be transcribed verbatim. A thematic analysis will be conducted on the transcripts of the focus groups and the data from the open-ended questions of the questionnaire data in the qualitative data management software Nvivo (QRS International Pty Ltd, 2022), in order to gain insight in the most important themes of the implementation of the intervention. Data will first be coded deductively (top-

down, data-driven) using the framework of Saunders et al. (2005), and additionally inductively (bottom-up, data-driven).

Baseline differences

Conditions will be compared on baseline sociodemographic characteristics and outcome measures, using one-way ANOVA’s for normally distributed continuous data, Kruskal-Wallis tests for non-normally distributed continuous data and ordinal data, and Chi-square tests for nominal data.

7. References

Balsamo, M., Cataldi, F., Carlucci, L., Padulo, C., & Fairfield, B. (2018). Assessment of late-life depression via self-report measures: a review. *Clinical Interventions in Aging*, *13*, 2021-2044. https://doi.org/10.2147/CIA.S178943

Barnett, A., van den Hoek, D., Barnett, D., & Cerin, E. (2016). Measuring moderate-intensity walking in older adults using the ActiGraph accelerometer. *BMC Geriatr*, *16*(1), 211. https://doi.org/10.1186/s12877-016-0380-5

Bento-Torres, N. V. O., Bento-Torres, J., Tomás, A. M., Costa, V. O., Corrêa, P. G. R., Costa, C. N. M., Jardim, N. Y. V., & Picanço-Diniz, C. W. (2017). Influence of schooling and age on cognitive performance in healthy older adults. *Brazilian journal of medical and biological research = Revista brasileira de pesquisas medicas e biologicas*, *50*(4), e5892-e5892. https://doi.org/10.1590/1414-431X20165892

Broadbent, D. E., Cooper, P. F., FitzGerald, P., & Parkes, K. R. (1982). The cognitive failures questionnaire (CFQ) and its correlates. *British journal of clinical psychology*, *21*(1), 1-16.

Buysse, D., Yu, L., Moul, D., Germain, A., Stover, A., Dodds, N., Johnston, K., Shablesky-Cade, M., & Pilkonis, P. (2010). Development and Validation of Patient-Reported Outcome Measures for Sleep Disturbance and Sleep-Related Impairments. *Sleep*, *33*, 781-792. https://doi.org/10.1093/sleep/33.6.781

Carson, N., Leach, L., & Murphy, K. J. (2018). A re-examination of Montreal Cognitive Assessment (MoCA) cutoff scores. *Int J Geriatr Psychiatry*, *33*(2), 379-388. https://doi.org/10.1002/gps.4756

Choi, L., Liu, Z., Matthews, C. E., & Buchowski, M. S. (2011). Validation of accelerometer wear and nonwear time classification algorithm. *Med Sci Sports Exerc*, *43*(2), 357-364. https://doi.org/10.1249/MSS.0b013e3181ed61a3

Craig, C. L., Marshall, A. L., Sjöström, M., Bauman, A. E., Booth, M. L., Ainsworth, B. E., Pratt, M., Ekelund, U., Yngve, A., Sallis, J. F., & Oja, P. (2003). International physical activity questionnaire: 12-country reliability and validity. *Med Sci Sports Exerc*, *35*(8), 1381-1395. https://doi.org/10.1249/01.Mss.0000078924.61453.Fb

De Jong Gierveld, J., & Van Tilburg, T. (2008). De ingekorte schaal voor algemene, emotionele en sociale eenzaamheid. *Tijdschrift voor gerontologie en geriatrie*, *39*(1), 4-15.

De Jong Gierveld, J., & Van Tilburg, T. (2010). The De Jong Gierveld short scales for emotional and social loneliness: tested on data from 7 countries in the UN generations and gender surveys. *European Journal of Ageing*, *7*(2), 121-130. https://doi.org/10.1007/s10433-010-0144-6

Galvin, J. E., Tolea, M. I., & Chrisphonte, S. (2021). The Cognitive & Leisure Activity Scale (CLAS): A new measure to quantify cognitive activities in older adults with and without cognitive impairment. *Alzheimer's & Dementia: Translational Research & Clinical Interventions*, *7*(1), e12134. https://doi.org/10.1002/trc2.12134

Hoffman, L., & Walters, R. W. (2022). Catching up on multilevel modeling. *Annual Review of Psychology*, *73*, 659-689. https://doi.org/10.1146/annurev-psych-020821-103525

IBM Corp. (2021). IBM SPSS Statistics for Windows. In (Vol. Version 28.0). Armonk, NY: IBM Corp.

Kempen, G., & van Eijk, L. (1995). The psychometric properties of the SSL12-I, a short scale for measuring social support in the elderly. *Social Indicators Research*, *35*, 303-312. https://doi.org/10.1007/BF01079163

Köhler, S., Thomas, A. J., Barnett, N. A., & O'Brien, J. T. (2010). The pattern and course of cognitive impairment in late-life depression. *Psychol Med*, *40*(4), 591-602. https://doi.org/10.1017/s0033291709990833

Marent, P.-J., Vangilbergen, A., Chastin, S., Cardon, G., van Uffelen, J., G. Z., , & Beeckman, M. (2021). Conceptualization of A Cognitively Enriched Walking Program For Older Adults: A Co-Design Study With Experts and End Users [PREPRINT]. *BMC Geriatrics*. https://doi.org/10.21203/rs.3.rs-601900/v1 (available at Research Square [https://doi.org/10.21203/rs.3.rs-601900/v1])

Pellicciari, L., Chiarotto, A., Giusti, E., Crins, M. H. P., Roorda, L. D., & Terwee, C. B. (2021). Psychometric properties of the patient-reported outcomes measurement information system scale v1.2: global

health (PROMIS-GH) in a Dutch general population. *Health and quality of life outcomes*, *19*(1), 226. https://doi.org/10.1186/s12955-021-01855-0

Ponds, R., van Boxtel, M. P. J., & Jolles, J. (2006). De Cognitive Failure Questionnaire als maat voor subjectief cognitief functioneren. *Tijdschrift voor neuropsychologie*, *1*(2), 37-45.

QRS International Pty Ltd. (2022). Nvivo. In (Vol. Version 1.7.1.).

Sahakian, B. J., & Owen, A. M. (1992). Computerized assessment in neuropsychiatry using CANTAB: discussion paper. *J R Soc Med*, *85*(7), 399-402.

Sarkisian, C. A., Steers, W. N., Hays, R. D., & Mangione, C. M. (2005). Development of the 12-item Expectations Regarding Aging Survey. *Gerontologist*, *45*(2), 240-248. https://doi.org/10.1093/geront/45.2.240

Saunders, R. P., Evans, M. H., & Joshi, P. (2005). Developing a process-evaluation plan for assessing health promotion program implementation: a how-to guide. *Health Promot Pract*, *6*(2), 134-147. https://doi.org/10.1177/1524839904273387

Tennant, R., Hiller, L., Fishwick, R., Platt, S., Joseph, S., Weich, S., Parkinson, J., Secker, J., & Stewart-Brown, S. (2007). The Warwick-Edinburgh Mental Well-being Scale (WEMWBS): development and UK validation. *Health and quality of life outcomes*, *5*, 63-63. https://doi.org/10.1186/1477-7525-5-63

Terwee, C. B., Roorda, L. D., de Vet, H. C., Dekker, J., Westhovens, R., van Leeuwen, J., Cella, D., Correia, H., Arnold, B., Perez, B., & Boers, M. (2014). Dutch-Flemish translation of 17 item banks from the patient-reported outcomes measurement information system (PROMIS). *Qual Life Res*, *23*(6), 1733-1741. https://doi.org/10.1007/s11136-013-0611-6

van Eijk, L. M., Kempen, G. I., & van Sonderen, F. L. (1994). [A short scale for measuring social support in the elderly: the SSL12-I]. *Tijdschr Gerontol Geriatr*, *25*(5), 192-196. (Een korte schaal voor het meten van sociale steun bij ouderen: de SSL12-I.)

Zygouris, S., & Tsolaki, M. (2015). Computerized cognitive testing for older adults: a review. *American Journal of Alzheimer's Disease & Other Dementias®*, *30*(1), 13-28. https://doi.org/10.1177/1533317514522852
