# Supplementary material for: Walking towards psychosocial well-being? Unveiling psychosocial impacts of a group-based walking program with and without cognitive enrichment in older adults—a mixed-methods randomized controlled trial
Source: PeerJ. 2026 Jan 22;14:e20569. doi: 10.7717/peerj.20569 (PMC12832057; doi:10.7717/peerj.20569)
Supplement: Supplemental Information 9 [file peerj-14-20569-s009.pdf]

CONSORT Reporting checklist for randomized controlled trials

		Reporting Item	Page Number
**Title and Abstract**			
Title	#1a	Identification as a randomized trial in the title.	1
Abstract	#1b	Structured summary of trial design, methods, results, and conclusions	2
**Introduction**			
Background and objectives	#2a	Scientific background and explanation of rationale	3-6
Background and objectives	#2b	Specific objectives or hypothesis	6
**Methods**			
Trial design	#3a	Description of trial design (such as parallel, factorial) including allocation ratio.	6
Trial design	#3b	Important changes to methods after trial commencement (such as eligibility criteria), with reasons	n/a
Participants	#4a	Eligibility criteria for participants	6-7
Participants	#4b	Settings and locations where the data were collected	6
Interventions	#5	The experimental and control interventions for each group with sufficient details to allow replication, including how and when they were actually administered	7-8
Outcomes	#6a	Completely defined prespecified primary and secondary outcome measures, including how and when they were assessed	8-9
Sample size	#7a	How sample size was determined.	7
Sample size	#7b	When applicable, explanation of any interim analyses and stopping guidelines	n/a
Randomization - Sequence generation	#8a	Method used to generate the random allocation sequence.	7
Randomization - Sequence generation	#8b	Type of randomization; details of any restriction (such as blocking and block size)	7
Randomization - Allocation concealment mechanism	#9	Mechanism used to implement the random allocation sequence (such as sequentially numbered containers), describing any steps taken to conceal the sequence until interventions were assigned	7
Randomization - Implementation	#10	Who generated the allocation sequence, who enrolled participants, and who assigned participants to interventions	7
Blinding	#11a	If done, who was blinded after assignment to interventions (for example, participants, care providers, those assessing outcomes) and how.	7
Blinding	#11b	If relevant, description of the similarity of interventions	7-8
Statistical methods	#12a	Statistical methods used to compare groups for primary and secondary outcomes	10
Statistical methods	#12b	Methods for additional analyses, such as subgroup analyses and adjusted analyses	10

Outcomes	#6b	Any changes to trial outcomes after the trial commenced, with reasons	n/a
**Results**			
Participant flow diagram (strongly recommended)	#13a	For each group, the numbers of participants who were randomly assigned, received intended treatment, and were analysed for the primary outcome	fig. 1
Participant flow	#13b	For each group, losses and exclusions after randomization, together with reason	fig. 1
Recruitment	#14a	Dates defining the periods of recruitment and follow-up	6
Recruitment	#14b	Why the trial ended or was stopped	n/a
Baseline data	#15	A table showing baseline demographic and clinical characteristics for each group	table 1
Numbers analysed	#16	For each group, number of participants (denominator) included in each analysis and whether the analysis was by original assigned groups	Fig 1; Table 3, p. 11
Outcomes and estimation	#17a	For each primary and secondary outcome, results for each group, and the estimated effect size and its precision (such as 95% confidence interval)	11-13
Outcomes and estimation	#17b	For binary outcomes, presentation of both absolute and relative effect sizes is recommended	n/a
Ancillary analyses	#18	Results of any other analyses performed, including subgroup analyses and adjusted analyses, distinguishing pre-specified from exploratory	11-13
Harms	#19	All important harms or unintended effects in each group (For specific guidance see CONSORT for harms)	n/a
**Discussion**			
Limitations	#20	Trial limitations, addressing sources of potential bias, imprecision, and, if relevant, multiplicity of analyses	14-16
Interpretation	#22	Interpretation consistent with results, balancing benefits and harms, and considering other relevant evidence	13-17
Registration	#23	Registration number and name of trial registry	6
Generalisability	#21	Generalisability (external validity, applicability) of the trial findings	15-16
**Other information**			
Interpretation	#22	Interpretation consistent with results, balancing benefits and harms, and considering other relevant evidence	17
Registration	#23	Registration number and name of trial registry	6
Protocol	#24	Where the full trial protocol can be accessed, if available	6
Funding	#25	Sources of funding and other support (such as supply of drugs), role of funders	17

Notes:

- 16: Fig 1; Table 3, p. 15, p.16 The CONSORT checklist is distributed under the terms of the Creative Commons Attribution License CC-BY. This checklist was completed on 29. November 2024 using https://www.goodreports.org/, a tool made by the EQUATOR Network in collaboration with Penelope.ai
